# Parkinson’s disease and schizophrenia interactomes contain temporally distinct gene clusters underlying comorbid mechanisms and unique disease processes

**DOI:** 10.1038/s41537-024-00439-3

**Published:** 2024-02-27

**Authors:** Kalyani B. Karunakaran, Sanjeev Jain, Samir K. Brahmachari, N. Balakrishnan, Madhavi K. Ganapathiraju

**Affiliations:** 1 Supercomputer Education and Research Centre, Indian Institute of Science, Bangalore, India; 2 Institute for the Advanced Study of Human Biology, Kyoto University, Kyoto, Japan; 3 National Institute of Mental Health and Neuro-Sciences (NIMHANS), Bangalore, India; 4 Academy of Scientific and Innovative Research, CSIR-4PI Bangalore, India; 5 Department of Computer Science, Carnegie Mellon University Qatar, Doha, Qatar; 6 Department of Biomedical Informatics, School of Medicine, University of Pittsburgh, Pittsburgh, PA USA

**Keywords:** Schizophrenia, Genetics of the nervous system

## Abstract

Genome-wide association studies suggest significant overlaps in Parkinson’s disease (PD) and schizophrenia (SZ) risks, but the underlying mechanisms remain elusive. The protein-protein interaction network (‘interactome’) plays a crucial role in PD and SZ and can incorporate their spatiotemporal specificities. Therefore, to study the linked biology of PD and SZ, we compiled PD- and SZ-associated genes from the DisGeNET database, and constructed their interactomes using BioGRID and HPRD. We examined the interactomes using clustering and enrichment analyses, in conjunction with the transcriptomic data of 26 brain regions spanning foetal stages to adulthood available in the BrainSpan Atlas. PD and SZ interactomes formed four gene clusters with distinct temporal identities (Disease Gene Networks or ‘DGNs’1-4). DGN1 had unique SZ interactome genes highly expressed across developmental stages, corresponding to a neurodevelopmental SZ subtype. DGN2, containing unique SZ interactome genes expressed from early infancy to adulthood, correlated with an inflammation-driven SZ subtype and adult SZ risk. DGN3 contained unique PD interactome genes expressed in late infancy, early and late childhood, and adulthood, and involved in mitochondrial pathways. DGN4, containing prenatally-expressed genes common to both the interactomes, involved in stem cell pluripotency and overlapping with the interactome of 22q11 deletion syndrome (comorbid psychosis and Parkinsonism), potentially regulates neurodevelopmental mechanisms in PD-SZ comorbidity. Our findings suggest that disrupted neurodevelopment (regulated by DGN4) could expose risk windows in PD and SZ, later elevating disease risk through inflammation (DGN2). Alternatively, variant clustering in DGNs may produce disease subtypes, e.g., PD-SZ comorbidity with DGN4, and early/late-onset SZ with DGN1/DGN2.

## Introduction

Parkinson’s disease (PD) has historically been attributed to a hypodopaminergic state in the nigrostriatal pathway characterized by reduced levels of dopamine compared to baseline^1^. In contrast, schizophrenia (SZ) has been linked to a transient hyperdopaminergic state characterized by enhanced levels of dopamine in the mesolimbic pathway^1^. PD often begins in the older population, and affects 1% of the individuals above 60 years and 5% among those above 85 years^2^, whereas SZ occurs during early adulthood most often before 25 years of age, and has a lifetime risk of 1%^3^.

Co-occurrence of PD and SZ symptoms has often been observed in Lewy body dementia (LBD)^4–6^. In addition, PD patients, including drug-naïve ones, experience cognitive decline and psychotic symptoms such as delusions and visual hallucinations in early PD stages and multimodal hallucinations in later stages^7^. Conversely, Parkinsonism has often been reported in drug-naïve SZ patients^8^. Drugs used to treat PD (e.g. levodopa) increase the risk of developing psychotic symptoms^9,10^. Drugs used to treat SZ (e.g. flupentixol) are, in turn, associated with a complex array of symptoms linked to basal ganglia dysfunction including Parkinsonism, dystonia and dyskinesia^11^. For example, when treated for their SZ-like symptoms with such antipsychotic drugs, patients with mutations in the *PLA2G6* gene develop severe extrapyramidal symptoms^12^. Finally, people with SZ have increased risk of developing PD^1^. Such an extensive intersection between their pathophysymptomatologies could be explained by a common genetic basis for PD and SZ, as corroborated by the identification of genetic variants exposing to both Parkinsonism and psychotic symptoms^12–14^.

**Fig. 1:**
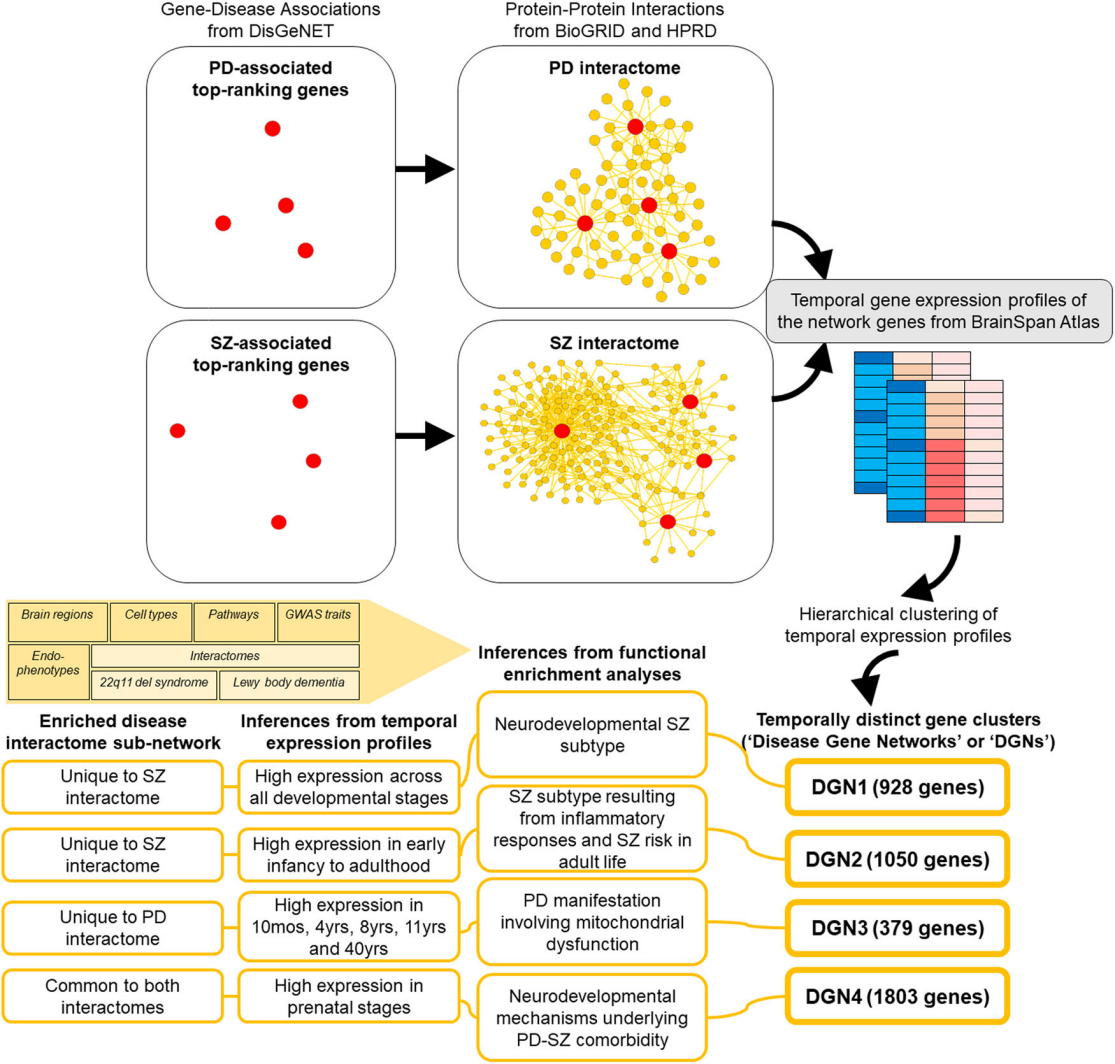
Flow chart depicting the methodology and the findings of the study. To examine the neurobiological underpinnings of PD and SZ, the study focused on disease interactome analysis. Genes associated with PD and SZ were compiled and used to construct the PD and SZ disease interactomes. Next, the temporal expression profiles of the genes in these networks were examined using hierarchical clustering to identify temporally distinct clusters. Four temporally distinct clusters were extracted from the disease interactomes (disease gene networks or ‘DGNs’ 1-4). Their temporal characteristics are listed under “inferences from temporal expression profiles”. Their enrichments for specific sub-networks in the disease interactomes are listed under “enriched disease interactome sub-networks”. Finally, the four DGNs were characterized based on their enrichment patterns in specific brain regions, cell types, pathways, GWAS traits, and disease endophenotypes curated from literature. Their relationships with the interactomes of 22q11.2 deletion syndrome and Lewy body dementia, in which affected individuals develop both psychosis and Parkinsonian symptoms, were also examined. The overall findings from these analyses are listed under “inferences from functional enrichment analyses”.

Genome-wide association studies (GWAS)^15–19^ and meta-analyses^20–24^ have helped identify several risk loci shared between the disorders. However, further analyses are required to elucidate the molecular mechanisms by which the genetic basis translates to the comorbidity of PD and SZ. We used the framework of the protein-protein interaction (PPI) network or the ‘interactome’ to explore the higher-order relationships in the genetic structures of PD and SZ^25–29^. We expect interactome-driven effects to play crucial roles in their biology^25,30,31^. Moreover, since the interactome analysis framework can incorporate spatiotemporal specificities^32^, it may help understand the linked biology of PD and SZ^1–3,33^. Therefore, we systematically analysed the interactomes of genes associated with PD and SZ in conjunction with spatiotemporal transcriptomic data to identify the neurobiological mechanisms underlying the two disorders. The methodology and the overall findings of the study can be seen in Fig. 1.

## Results

### Construction of PD and SZ interactomes

We identified 100 top-ranking PD and SZ associated genes, including those causally contributing to the diseases from DisGeNET based on their gene-disease association (GDA) scores (Supplementary Note S1 and Supplementary Data File S1)^34^. The GDA scores are computed based on the number of publications and curated human/model organism databases reporting the association. For the construction of PD and SZ interactomes, we compiled the immediate neighbors of the genes in the network of human PPIs using the Human Protein Reference Database (HPRD)^35^ and the Biological General Repository for Interaction Datasets (BioGRID) (Supplementary Data File S1)^36^. The PD and SZ interactomes contained 3200 and 2662 genes, respectively. We took a systematic approach to analyze the spatiotemporal expression patterns of the interactomes. The two interactomes share 1232 genes, a statistically significant overlap (*p*-value = 2.88E-310) with 2.6-fold enrichment, based on the distribution of PD and SZ interactome genes in a universal set of 17,992 genes from the BioGRID and HPRD databases. To validate the overlap between PD and SZ interactomes, we compared it to their respective overlaps with the interactomes of the top-100 genes linked to four other disorders (collected from the DisGeNET database), namely, Alzheimer’s disease, celiac disease, Crohn’s disease, and peroxisomal disorder. We selected these disorders for their non-overlapping genetic epidemiology with PD and SZ and their impact on tissues other than the brain (in the case of the latter three disorders). The inclusion of Alzheimer’s disorder, a neurological disorder, could help capture unique features of PD and SZ as brain disorders. We employed hypergeometric testing and applied the Benjamini-Hochberg correction for multiple hypotheses. This analysis confirmed exclusive overlaps between the PD and SZ interactomes; the PD interactome overlapped significantly only with the SZ interactome (Supplementary Fig. S1a), and vice versa (Supplementary Fig. S1b), when considered alongside these selected disorders.

### Identification of gene clusters with distinct temporal expression profiles

**Fig. 2:**
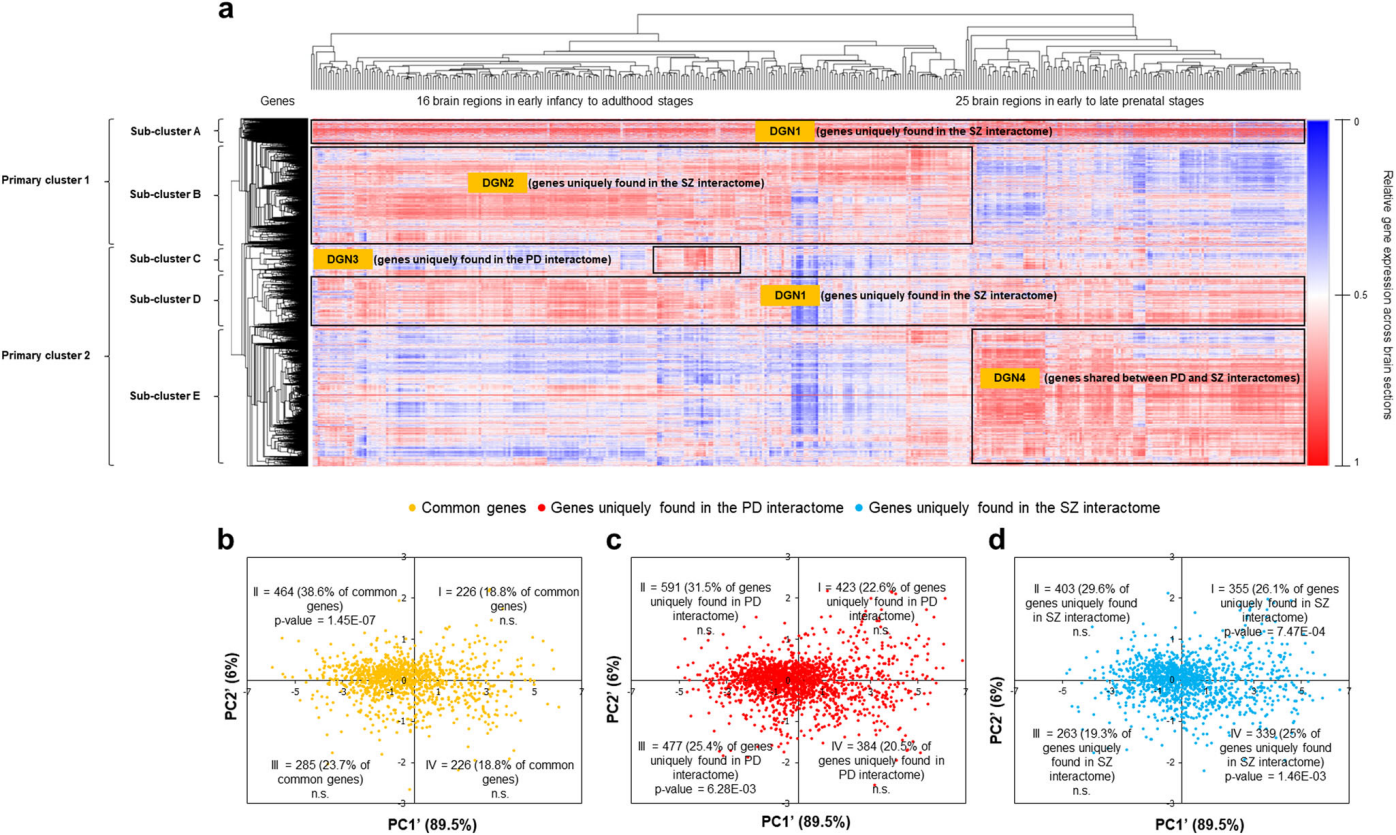
Genes in PD and SZ interactomes split into four clusters based on their temporal expression patterns. **a** The figure shows the two spatiotemporal clusters – corresponding to 16 brain regions in early infancy to adulthood stages (left) and 25 brain regions in early prenatal to late prenatal stages (right) – on the horizontal axis. The relative expression of 4436 out of the 4629 genes present in the PD and SZ interactomes across 407 spatiotemporal points was hierarchically clustered. Clustering was performed on log-transformed RPKM (i.e. Reads Per Kilobase per Million mapped reads) values using the hierarchical clustering method with average linkage. The dendrograms were derived from the clustering analysis based on the computation of Pearson correlation coefficients between the data points. The clustered heat map was created using the Morpheus software. Two primary gene clusters can be seen on the vertical axis, which can be further subdivided into five sub-clusters (A-E) showing distinct temporal profiles. These sub-clusters were labelled as Disease Gene Networks or ‘DGNs’1-4 based on their preferential enrichment for proteins uniquely found in the PD/SZ interactomes or shared between both the interactomes. **b**–**d** PCA was performed with the expression profiles of the genes belonging to PD and SZ interactomes in 2 brain regions in various developmental stages (A1C-24pcw, A1C-25pcw, A1C-37pcw and AMY-21pcw). A matrix with 4436 genes (rows) and 4 spatiotemporal conditions (columns) was constructed out of log-transformed RPKM values. Unit variance scaling was applied across this matrix. Singular value decomposition (SVD) with imputation was used to extract principal components (PCs). Component scores corresponding to PC1’ and PC2’ explaining 89.5% and 6% of the total variance were plotted along X and Y axes respectively. Component scores and quadrant-wise enrichment of genes (**b**) shared between PD and SZ interactomes, (**c**) uniquely found in the PD interactome, and (**d**) uniquely found in the SZ interactome. The percentage of genes from each of these gene sets found in each of the 4 quadrants is also shown. Note that these analyses were performed using data collected from a single source (Human Developmental Transcriptome, BrainSpan Atlas).

We examined the expression patterns of the genes in the PD and SZ interactomes using the developmental transcriptome data available in the BrainSpan Atlas^37^, spanning 407 spatiotemporal coordinates, specifically, 26 brain structures at ages ranging from 8 post-conception weeks (pcw; prenatal stage) to 40 years (adulthood) (see Methods and Supplementary Data File S2 for the data matrix of genes and brain samples). We jointly examined the spatial and temporal aspects of the brain samples using hierarchical clustering analysis. The samples formed distinct clusters solely based on their temporal stages, specifically, segregating into two groups: those from early to late prenatal stages and those from early infancy to adulthood stages (see top horizontal axis in Fig. 2a).

Along the vertical axis, we identified two primary gene clusters and five sub-clusters (A-E), with A and B belonging to the first primary cluster, and C, D and E belonging to the second primary cluster (Fig. 2a and Supplementary Data File S3 for hierarchical clustering output matrix). Sub-clusters A and D, which belong to different primary clusters and together contain 928 genes, displayed consistent high expression across all ages, and were enriched for genes unique to the SZ interactome (FDR-adjusted *p*-value = 0.041, where FDR is false discovery rate). Sub-cluster B, containing 1,050 genes, showed high expression in 16 brain regions from early infancy to adulthood and was enriched for SZ-interactome-specific genes (FDR-adjusted *p*-value = 0.018). Sub-cluster C, containing 379 genes, exhibited elevated expression at 10 months, 4 years, 8 years, 11 years, and 40 years, with enrichment for PD-interactome-specific genes (FDR-adjusted *p*-value = 2.62E-12). Sub-cluster E, containing 1803 genes, showed high expression during prenatal stages and enrichment for genes shared between the PD and SZ interactomes (FDR-adjusted *p*-value = 1.44E-05). The enrichments were computed using a hypergeometric test. A hypergeometric *p*-value that remained less than 0.05, after multiple hypotheses correction using the Benjamini-Hochberg method for FDR estimation, was considered to be statistically significant.

Principal component analysis (PCA) identified a prominent division separating genes shared between PD and SZ interactomes (quadrant II in Fig. 2b) and unique PD interactome-specific genes (quadrant III in Fig. 2c) from SZ interactome-specific genes (quadrants I and IV in Fig. 2d; see Supplementary Note S2 for details of PCA and Supplementary Data File S4 for PCA output matrices). This reflected the primary gene clusters obtained from hierarchical clustering (Fig. 2a), where the first primary cluster contained sub-clusters A and B enriched for SZ interactome-specific genes, while the second primary cluster contained sub-clusters C and E enriched for PD interactome-specific genes and shared genes, respectively. Since the 928 genes in sub-clusters A and D, identified through hierarchical clustering, exhibited significant enrichment exclusively in quadrant I of the PC plot in Fig. 2d (FDR-adjusted *p*-value = <2.2E-16), and considering that PC2, being the only possible axis of transcriptomic variation between sub-clusters A and D, accounted for only 6% variance, distinguishing between these two sub-clusters as distinct entities became unnecessary. Consequently, we treated these sub-clusters, which contained unique SZ interactome genes expressed across developmental stages, as a unified group named DGN1, where DGN stands for ‘Disease Gene Network’. Sub-cluster B, containing unique SZ interactome genes expressed from infancy to adulthood, was labelled DGN2, while sub-cluster C, containing unique PD interactome genes expressed at specific temporal stages, was labelled DGN3. Sub-cluster E, containing genes shared between the interactomes during prenatal stages, was labelled DGN4. Lastly, in line with the disorder affiliations of the DGNs and PCA quadrants, we observed statistically significant enrichments of DGN1, DGN2, DGN3, and DGN4 in PCA quadrants I, IV, III, and II, respectively (Supplementary Fig. S2). Moreover, the disorder interactome genes in corresponding DGNs and quadrants significantly contributed to these enrichments. Specifically, 36.5% of quadrant I genes intersecting with DGN1 (123 out of 337 genes) were unique SZ interactome genes (FDR-adjusted *p*-value = 5.21E-53) as were 38.6% of quadrant IV genes intersecting with DGN2 (177 of 458 genes; FDR-adjusted *p*-value = 1.27E-106). In addition, 64.8% of quadrant III genes intersecting with DGN3 were unique PD interactome genes (107 of 165 genes; FDR-adjusted *p*-value = 5.08E-62), and 34.9% of quadrant II genes intersecting with DGN4 were shared interactome genes (291 of 833 genes; FDR-adjusted *p*-value = 4.3E-200).

We additionally characterized the four DGNs according to their enrichment for brain cell types (based on marker genes compiled from ref. ^38^) and KEGG pathways (Supplementary Table S1 and Supplementary Table S2)^39^. DGN1 was enriched with excitatory cell markers (FDR-adjusted *p*-value 0.008), and with proteins related to the functioning of dopaminergic synapses (FDR-adjusted *p*-value 5E-08). DGN2 was enriched with oligodendrocyte markers (FDR-adjusted *p*-value 0.049), and proteins involved in T helper cell 17 (T_H_17) differentiation (FDR-adjusted *p*-value 1.45E-13). DGN3 was enriched with proteins in the proteasome complex (FDR-adjusted *p*-value 1.58E-09) (Supplementary Note S3). DGN4 was enriched with genes encoding cell cycle proteins (FDR-adjusted *p*-value 7.49E-12). Neither DGN3 nor DGN4 was enriched for molecular markers of cell types or neuronal signalling pathways. Thus, the PD and SZ interactomes contained four temporally and functionally distinct gene clusters.

### Regional specificities of the DGNs

Several studies indicate that spatial distribution of gene expression in various brain regions can be correlated with functional connectivity between them^40–45^. Based on this, we speculated that the differences in the pattern of the temporally defined DGNs in specific brain regions, may reveal the neural mechanisms driving the onset of symptoms in PD and SZ. We examined whether the DGNs detected from PD and SZ interactomes were enriched for expression in 22 postnatal human brain regions and 4 transitory foetal structures (lateral, medial and caudal ganglionic eminences, and rhombic lip; as available in BrainSpan Atlas (see Supplementary Methods))^46^. We then asked whether specific DGNs clustered together based on their regional specificities. In previous studies, using –log_10_ transformed *p*-values of enrichment for clustering analyses has helped derive cross-trait associations based on pathway enrichment among trait-associated genes^47^, and sub-groups of anxiety disorder subtypes based on the regional enrichment patterns of subtype-associated genes^48^. Therefore, the –log_10_ transformed p-values of regional enrichment were used as inputs for the clustering analysis. The log-transformed *p*-values were converted to normalized z-scores, and subjected to hierarchical clustering based on Pearson’s correlation coefficient and the average linkage method.

**Fig. 3:**
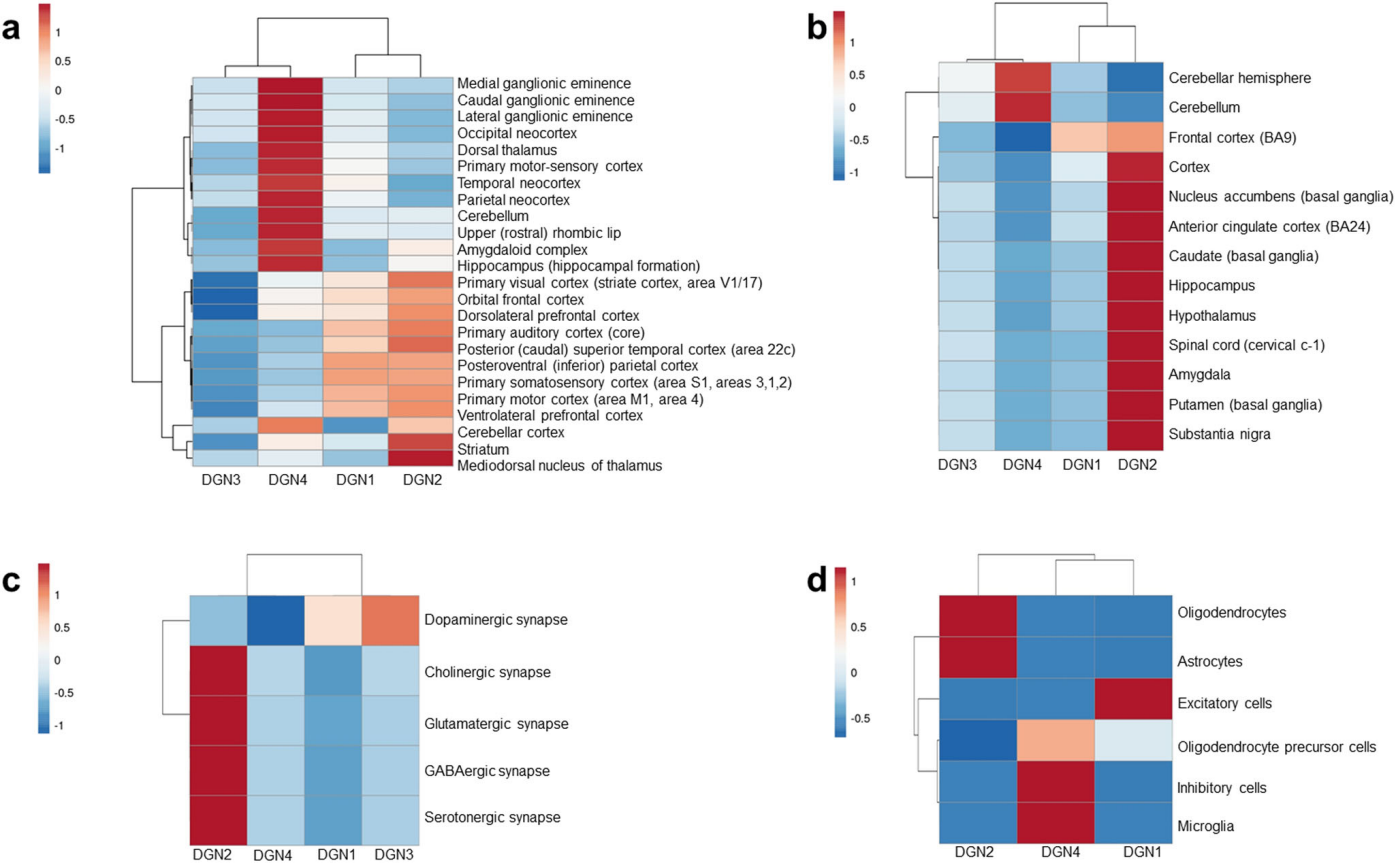
DGNs 1-4 showed differential enrichment patterns in specific brain regions, signalling pathways and brain cell types. The figures show the enrichment of the DGNs for genes (**a**) expressed across 26 brain regions compiled from BrainSpan Atlas having logRPKM > 2, (**b**) showing high/medium expression in 13 brain regions (Transcripts Per Million or TPM > 9) compiled from GTEx, (**c**) involved in five neuronal synaptic signalling pathways, i.e., cholinergic, dopaminergic, GABAergic, glutamatergic and serotonergic pathways compiled from the KEGG database, and (**d**) specifically expressed in 6 brain cell types compiled from a study by Lake et al.^38^ The statistical significance of each enrichment was computed as a *p*-value. All p-values were transformed to –log_10_p-values, and then assembled into a data matrix containing brain regions as rows and the DGNs as columns. Negative log transformation simplifies the scale, revealing patterns and significant results more visibly, with higher –log_10_ values signifying smaller *p*-values. Variations in enrichment are represented in the form of heat maps. Specifically, z-scores computed based on the inverse normal transformation of –log_10_ transformed p-values are shown. Clustering was performed using the hierarchical clustering method with average linkage. The dendrograms were derived from the clustering analysis based on the computation of Pearson correlation coefficients between the data points. The clustered heat map was created using the ClustVis software. Note that this analysis was performed with gene expression profiles of brain samples obtained from a single source (BrainSpan Atlas).

We observed two main DGN clusters (Fig. 3a and Supplementary Table S3 for enrichment results). The first cluster included DGN3 (unique PD) and DGN4 (shared), and the second cluster included DGN1 and DGN2 (both are unique to SZ). Two major brain region clusters further subdivided into four sub-clusters as shown in Fig. 3a. DGN1 and DGN2 showed enrichment in specific areas, including the orbital frontal, dorsolateral, and ventrolateral prefrontal cortices, distinguishing them from DGN3 and DGN4, which showed lower enrichment in these regions. On the other hand, DGN4 demonstrated enrichment in striatum, amygdala, hippocampus, cerebellum, and foetal tissues (ganglionic eminences and upper rhombic lip).

Next, we examined whether DGNs were enriched for expression in 13 postnatal human brain regions available in GTEx (see Supplementary Methods)^49^. Despite using a less diverse dataset, the two DGN clusters were preserved (Fig. 3b and Supplementary Table S4 for enrichment results). Consistent with Fig. 3a, we noted that the enrichment of frontal cortical regions in DGN1 and DGN2 segregated them from DGN3 and DGN4. Additionally, the enrichment of DGN3 and DGN4 in the cerebellum segregated them from DGN1 and DGN2 (Fig. 3b), indicating larger involvement of motor control deficits in the former.

Although DGN1 and DGN2 both contained genes unique to the SZ interactome and clustered together, they exhibited several notable regional patterns that differentiated them. One, in the analysis with BrainSpan Atlas data (Fig. 3a), DGN2 showed enrichment in the subcortical structures of the limbic circuit (amygdala, hippocampus, and striatum), as well as the connected structures (mediodorsal thalamic nucleus and cerebellar cortex), distinguishing it from DGN1. This was also supported by GTEx data (Fig. 3b), in which the enrichment of DGN2 in cortical, subcortical, and midbrain structures of the limbic circuit differentiated it from DGN1, namely, the anterior cingulate cortex, amygdala, hippocampus, caudate nucleus, putamen, nucleus accumbens, hypothalamus and substantia nigra. DGN2 showed exclusive enrichment for genes associated with anxiety and depressive disorders (Supplementary Note S4), whose biology is mediated by the limbic structures^50^, as distinct from DGN1. The anxiety and depressive disorder associated genes were themselves highly enriched in these structures (Supplementary Note S4). Hence, DGN2 appears to be a SZ subtype potentially associated with affective disorganization, and anxiety and depression^51,52^. Two, DGN1 showed higher (or exclusive) enrichment than DGN2 in ventral telencephalic structures such as the ganglionic eminences that populate limbic circuit regions and form cortical interneurons, hinting at the neurodevelopmental underpinnings of its constituent SZ interactome genes.

The separation of the cluster containing DGN1 and DGN2, from the cluster of DGN3 and DGN4, was also observed with a prenatal microarray dataset available in the BrainSpan Atlas (Supplementary Fig. S3 and Supplementary Table S5 for enrichment results)^46^. Although this independent dataset supports the split between the DGNs, further analysis will be required to understand the implications of the prenatal regional specificities driving this division.

Altogether, DGN1 consistently clustered with DGN2; and DGN3 with DGN4; based on regional expression profiles of foetal and adult brain structures, extracted from three independent datasets. We suggest that DGN1 and DGN2 may represent two putative SZ subtypes, characterized by differences in expression across specific regions of the brain. It is notable that pathway analysis using WebGestalt^53^ recapitulated the clustering of DGN1 with DGN2 and DGN3 with DGN4 (Supplementary Fig. S4 and Supplementary Table S2 for enrichment results), similar to the clustering pattern observed based on their regional specificities. This analysis also revealed the difference between the suggested SZ subtypes, which will be discussed in a later section.

### Biological characterization of DGN4 containing genes shared between PD and SZ interactomes

**Fig. 4:**
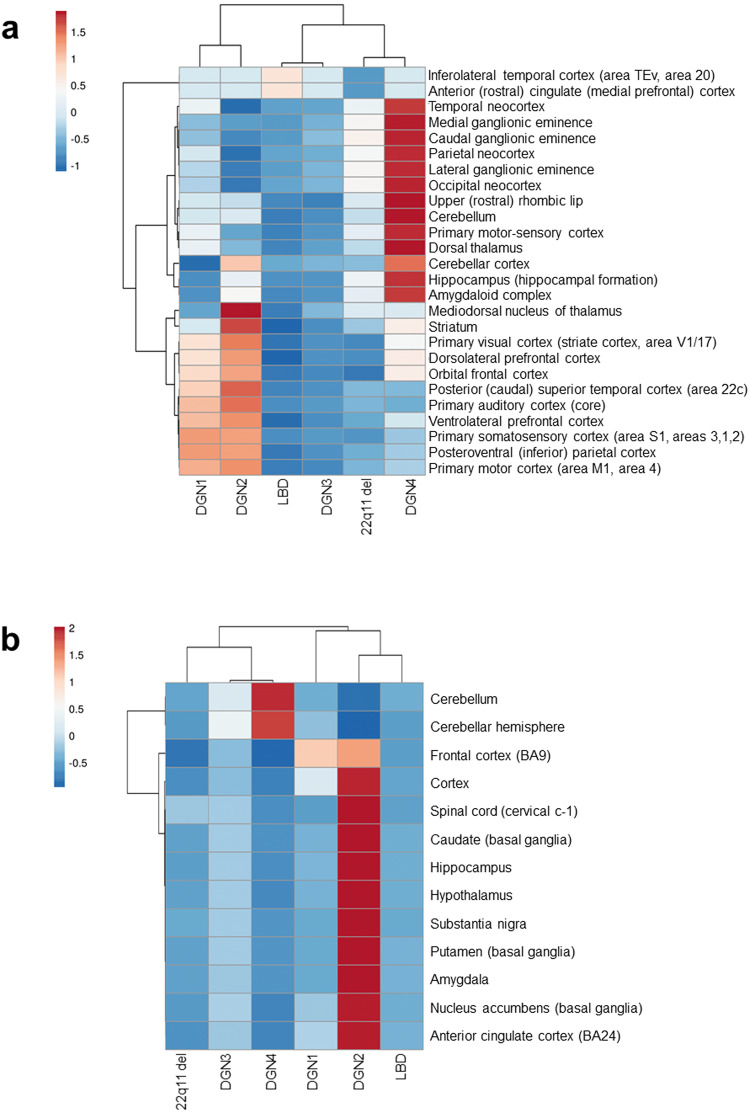
Lewy body disease and 22q11 deletion syndrome interactomes clustered with specific DGNs. The enrichment of the genes belonging to DGNs, and the interactomes of GWAS genes associated with Lewy body disease (LBD) and genes differentially expressed in SZ patients with 22q11 deletion (22q11 del), in (**a**) 26 brain regions from BrainSpan Atlas and (**b**) 13 brain regions in GTEx. The statistical significance of each region-wise enrichment was computed as a *p*-value. All p-values were transformed to –log_10_p-values, and then assembled into a data matrix containing brain regions as rows and the DGNs as columns. Negative log transformation simplifies the scale, revealing patterns and significant results more visibly, with higher –log_10_ values signifying smaller *p*-values. Variations in region-wise enrichment have been represented in the form of heat maps. Specifically, z-scores computed based on the inverse normal transformation of –log_10_ transformed *p*-values are shown in (**a**, **b**). Clustering was performed using the hierarchical clustering method with average linkage. The dendrograms were derived from the clustering analysis based on the computation of Pearson correlation coefficients between the data points. The clustered heat map was created using the ClustVis software. Note that the results shown in (**a**, **b**) were obtained using data from a single source, i.e., BrainSpan Atlas in the case of (**a**) and GTEx in the case of (**b**).

We compared the DGNs with the genes associated with 22q11 deletion syndrome, in which Parkinsonian and psychotic symptoms often co-exist^17,54^, to detect plausible overlaps. Specifically, we checked the enrichment of the four DGNs in the interactome of the genes differentially expressed in SZ patients with 22q11 deletion syndrome compared to healthy subjects^55^ (Supplementary Methods and Supplementary Data File S5). DGN4, which contained genes shared between PD and SZ interactomes, alone showed enrichment in this new interactome (Supplementary Note S5)^55^. DGN4 also clustered with the 22q11 deletion interactome, based on their shared enrichment patterns (Supplementary Note S5) in the foetal structures present in BrainSpan Atlas (Fig. 4a) and GTEx (Fig. 4b); see Supplementary Table S6 and Supplementary Table S7 for the respective enrichment results.

The highest enrichment for transitory foetal structures including the ganglionic eminences and the rhombic lip (that populates the cerebellum) was shown by DGN4 (Fig. 3a), suggesting the involvement of the genes shared between PD and SZ interactomes in the neurodevelopmental processes. Enrichment analysis with KEGG pathways^39^ showed that DGN4 was highly enriched in pathways regulating pluripotency of stem cells and developmental pathways such as Notch and Hippo (Supplementary Fig. S4 and Supplementary Table S2 for enrichment results) (at FDR-adjusted *p*-value < 0.05). Collectively, this suggested a potential role for DGN4 in regulating the differentiation and maturation of neural stem cells, cell fate and organ development, processes possibly critical to the shared neurodevelopmental aspects of PD and SZ. Pathway analysis also showed that the genes in DGN4 were highly enriched for cancer-related pathways, indicating their roles in essential cellular functions (Supplementary Fig. S4), and protein complexes involved in mRNA splicing and translation. In addition to these enriched pathways, we checked the presence of topological modules in DGN4. Topological modules are genes that are more highly interconnected among themselves than with the rest of the genes in a protein interaction network^32^. Two modules from DGN4 were enriched with proteins involved in peptide chain elongation (66 and 5 genes in modules, FDR-adjusted *p*-value < 1E-13 and FDR-adjusted *p*-value = 4.87E-05, respectively), and two were enriched with proteins involved in mRNA splicing (14 and 50 genes in modules, FDR-adjusted *p*-value = 5.71E-13 and FDR-adjusted *p*-value = 3.27E-05, respectively). Additionally, two modules were enriched with proteins involved in translation initiation complex formation (FDR-adjusted p-value = 4.97E-10) and rRNA processing in the nucleolus and cytosol (FDR-adjusted *p*-value = 8E-07). The identification of these modules suggested gene expression and protein synthesis as processes central to the understanding of PD and SZ comorbidity. Altogether, the evidence from transcriptomic, pathway and network module data suggested that DGN4 is implicated in neurodevelopment and neuroplasticity, and possibly risk of PD-SZ comorbidity.

### Biological characterization of DGN1 and DGN2 which are specific to schizophrenia

We examined the enrichment of the DGNs for brain cell type marker genes, which are specifically expressed in neuronal/non-neuronal cell populations of the prefrontal cortex (compiled from ref. ^38^; see Supplementary Methods). These included excitatory, inhibitory and microglial cells, astrocytes, oligodendrocytes and oligodendrocyte precursor cells. We observed two clusters (Fig. 3d and Supplementary Table S1 for enrichment results), namely, (i) DGN2, and (ii) DGN4 and DGN1.

The enrichment of DGN2 for genes expressed in oligodendrocytes and astrocytes, which mediate immune processes, separated it from DGN4 and DGN1. We also observed that DGN2 showed the highest significant enrichment for 5 clusters of 36 immune-related pathways including those involved in Epstein-Barr virus infection, T_H_17, T_H_1, and T_H_2 cell differentiation, rheumatoid arthritis (RA), inflammatory bowel disease, and Cushing’s syndrome (Supplementary Fig. S4). T_H_17-mediated immune processes are often perturbed in SZ patients^56,57^. Interestingly, these processes also regulate the functions of oligodendrocytes and astrocytes and play a critical role in neuroinflammation and neurodegeneration^58–61^.

Systemic lupus erythematosus (SLE) occurred as an immediate outgroup to the cluster of immune-related pathways and showed higher enrichment in DGN1 than DGN2 (Supplementary Fig. S4). We compiled a set of 431 SLE-associated genes and 263 RA-associated genes from the GWAS catalog^62^ for external validation. DGN2 showed exclusive enrichment for both RA (FDR-adjusted *p*-value = 0.02) and SLE (FDR-adjusted *p*-value = 1.28E-03) genes.

Interestingly, DGN2 was also enriched for SZ endophenotypes^63^ of sensorimotor gating that are tested using anti-saccade task (FDR-adjusted *p*-value = 0.047) and prepulse inhibition (FDR-adjusted *p*-value = 0.023) (Supplementary Fig. S5). Additionally, DGN2 showed higher enrichment for cholinergic, glutamatergic, GABAergic, and serotonergic signalling pathways, in contrast to DGN1 that showed enrichment for the dopaminergic signalling pathway, which clustered separately from the other pathways (Fig. 3c and Supplementary Table S8 for enrichment results). Together, this suggested that DGN2 could underlie SZ subtype with the involvement of limbic structures (as seen in Fig. 3a, b) and immune deficits contributing to SZ risk^64^; sensorimotor gating deficits are seen in SZ mouse models with immune deficits^65^.

The enrichment of DGN4 and DGN1 for oligodendrocyte precursor cell (OPC) markers separated them from DGN2 (Fig. 3d). This suggested the involvement of DGN4 and DGN1 in white matter defects arising from abnormal myelination, a process regulated by OPCs^66^. High enrichment for foetal structures helped distinguish DGN4 from the rest of the DGNs, and the putative SZ subtype DGN1 from the SZ subtype DGN2.

Overall, these results indicated that different biological processes may contribute to risk of SZ. DGN1 may represent a SZ subtype with neurodevelopmental underpinnings, whereas DGN2 may represent a subtype resulting from a later auto-immune/inflammatory response.

### Recapitulation of the DGNs with updated PPI, disease-associated genetic and brain-specific expression data

In our analyses, we compiled the PPIs of PD- and SZ-associated genes from BioGRID and HPRD, both of which are widely recognized and reliable sources of PPIs. Although HPRD has not been updated in recent years, it contains valuable PPI information. To ensure better data coverage, we additionally collected PPIs with medium (0.63) and high (0.7) confidence scores for the PD- and SZ-associated genes from the latest release of the HIPPIE database (v2.3)^67^. The expanded interactomes, incorporating BioGRID, HPRD and HIPPIE PPIs, successfully replicated four gene groups with temporal profiles and disease associations matching those of the DGNs, and they exhibited corresponding overlaps with the DGNs themselves (Supplementary Note S6 and Supplementary Fig. S6).

To ensure the tissue-specificity of PD and SZ interactomes, we intersected 15,331 genes expressed at > 1 nTPM (normalized transcripts per million) in the human brain, according to the Human Protein Atlas^68^, with our list of 4436 PD and SZ interactome genes (having expression profiles in the BrainSpan Atlas). This revealed that 3970 interactomes genes were expressed in the brain. Hierarchical clustering of their spatiotemporal profiles revealed four gene groups with distinct temporal patterns (Supplementary Fig. S7a): group-I, expressed throughout the stages and enriched for genes unique to the SZ interactome; prenatally expressed group-II, enriched for genes shared between PD and SZ interactomes; group-III, diverging from group-II due to expression in specific postnatal stages and enriched for genes unique to the PD interactome; and postnatally-expressed group-IV, enriched for genes unique to the SZ interactome (Supplementary Fig. S7b). Groups I-IV respectively exhibited overlaps with DGN1, DGN4, DGN3, and DGN2 (Supplementary Fig. S7c). This analysis confirms the consistent identification of these four gene groups with specific disorder associations from the PD and SZ interactomes, even when limited to brain-expressed genes. Note that group-III also overlapped with DGN4; the corresponding implications need to be explored further.

The evidence for gene-disease associations in the DisGeNET database is not limited to disease-associated human genetic variations. It additionally compiles information on genetic associations in model organisms, disease-related differential gene expression, post-translational modifications, and gene-dependent responses to therapeutics. To verify the validity of our findings, we curated 8 additional gene sets for SZ and PD from recent GWAS, and exome sequencing and family-based studies. Despite the varying designs of these studies, the identified genes, while not intersecting, often converge on similar biological themes^25^. The GWAS set for SZ included 120 genes mapped to loci of genome-wide significance (*P* < 5 × 10^−8^) and prioritized through fine-mapping, transcriptomic analysis, and functional genomic annotations (SZ Working Group of the Psychiatric Genomics Consortium)^69^. The exome-sequencing set for SZ included 10 genes linked to variants reaching exome-wide significance, i.e., *P* < 2.14 × 10^−6^ (Schizophrenia Exome Sequencing Meta-Analysis consortium)^70^. For PD, the GWAS sets included 29 genes mapped to loci achieving genome-wide significance and prioritized using Mendelian randomization^71^. The other sets included 21 genes in 11 loci of genome-wide significance^72^, a single gene (SNCA) linked to multiple loci of genome-wide significance in an independent study^73^, 11 genes mapped to loci achieving genome-wide significance, prioritized using co-localization analysis^74^, and 20 genes at loci of genome-wide or suggestive (*P* < 5 × 10^−5^) significance^75^. Lastly, 14 high-confidence PD-associated genes harbouring rare variants were collected based on the number of independent family-based studies reporting the variants and their associated functional evidence^76^. In this new collection, there were 81 PD-associated and 128 SZ-associated genes in total, of which 12 and 9 respectively intersected with our original PD and SZ gene sets (100 each) collected from DisGeNET. Therefore, combining genes from GWAS, exome-sequencing, and family-based studies with our original DisGeNET gene sets yielded 169 PD-associated genes and 219 SZ-associated genes. Upon extracting the PPIs of these genes from BioGRID and HPRD, we obtained PD and SZ interactomes containing 3871 and 4079 genes, respectively, with 2100 genes in common. The updated interactomes, integrating the latest genetic findings of PD and SZ, recapitulated the groups of genes following the temporal profiles and disease affiliations of DGN2 and DGN3 with high significance and that of DGN1 with modest significance (Supplementary Note S7 and Supplementary Fig. S8).

## Discussion

In this study, we combined network and transcriptomic analyses and identified four spatiotemporally distinct gene clusters from PD and SZ interactomes. Detailed biological characterization of the gene clusters suggested that one of them corresponds to mechanisms shared between PD and SZ, one to mechanisms unique to PD, and two others to subtypes of SZ. Our findings may help explain why symptoms of these syndromes often overlap and provide insights into the mechanisms underlying the relationship of PD and SZ. While in this study we highlight PD and SZ gene clusters as revealed through interactomics and transcriptomics, note that shared symptoms and comorbidity may stem from a complex interplay of genetic risk factors, individual genetic variability and environmental influences.

### Insights into disease mechanisms provided by the DGNs

Hierarchical clustering of PD and SZ interactome genes across the brain structures in different developmental phases revealed gene clusters with characteristic temporal identities (Fig. 2a). Across three independent expression datasets, DGN1 and DGN2 consistently showed a clear distinction from DGN3 and DGN4 (Fig. 3a, b and Supplementary Fig. S3). In the former, this distinction was driven by high enrichment for frontal cortical regions, which was in line with the observation of grey matter deficits in frontal cortical structures in SZ patients^77^, and the impaired performance of SZ patients in cognitive tasks dependent on frontal cortical activity^78^. In the latter, the distinction was driven by high enrichment for structures in the basal ganglia-cerebellar circuit, along with their progenitor populations (ganglionic eminences and rhombic lip), and connected structures in the limbic circuit (amygdala and hippocampus). This finding was consistent with the hypoactivation of the basal ganglia-cerebellar circuit noted during motor timing tasks in PD patients^79,80^. Pathway enrichment profiles (Supplementary Fig. S4) also reflected the association of DGN1 with DGN2 and DGN3 with DGN4. Hence, these results suggest that dynamic interactions across different biological scales, namely, cellular mechanisms, neural circuitry and temporal specificities, underlie the DGNs.

DGN4, which contains genes shared by the PD and SZ interactomes, perhaps identifies the neurodevelopmental mechanisms regulating the comorbidity of the two disorders. The cluster was highly expressed in prenatal stages and showed the highest enrichment for expression in foetal structures such as the ganglionic eminences and the rhombic lip (Fig. 3a). It was also enriched for pathways affecting stem cell pluripotency, embryonic development (Supplementary Fig. S4), and contained topological modules of proteins mediating splicing and translation. Moreover, DGN4 exclusively overlapped with the interactome of genes associated with 22q11 deletion syndrome, in which PD and SZ symptoms co-exist^17,54^. The segregation of DGN4 with the 22q11 deletion interactome based on their high enrichment in foetal structures is insightful. Lastly, DGN4 did not show enrichment for molecular markers of cell types or neuronal signalling pathways. However, it displayed a modest enrichment for genes associated with the higher-level mental attribute, abstraction and mental flexibility (FDR-adjusted *p*-value = 0.034), compiled from a single study that examined the trait as one of eleven SZ endophenotypes^63,81,82^. Notably, this trait was significantly associated with loci containing neurodevelopmental genes^63^. Overall, combined evidence from transcriptomic, pathway and network analyses suggest the role of DGN4 in disease risk, neurodevelopment and neuroplasticity.

The genes found to be unique to the SZ interactome segregated into DGN1 and DGN2, based on their distinct temporal identities (Fig. 2a). Analysis with two independent datasets revealed that these DGNs shared similar enrichment profiles across a diverse range of brain areas (Fig. 3a, b). These included areas involved in visual and auditory information processing, planning and execution of movements, processing somatosensory and motor stimuli to allow skilled movement, auditory processing and speech comprehension, and vital aspects of decision-making such as cognitive flexibility, response inhibition and goal-appropriate response selection.

However, DGN1 and DGN2 differed in some aspects. First, they showed differential enrichment in subcortical limbic areas and embryonic cells populating the ventral telencephalon, respectively. Second, in DGN1, there was enrichment for genes expressed in embryonic structures such as the ganglionic eminences (Fig. 3a) and marker genes of OPCs (Fig. 3d) whose dysregulation may lead to the white matter defects seen in syndromes of both PD and SZ^83,84^. Major white matter pathways that emerge during critical neurodevelopmental stages (e.g., projection, commissural, thalamocortical and association pathways between 3 and 32 pcw)^85^ may be particularly susceptible to disease-associated genetic and environmental vulnerabilities.

Third, multiple lines of evidence indicated that DGN2 may represent a SZ subtype in which immune insults in early life led to sensorimotor gating defects in adulthood. DGN2 showed the highest enrichment for a large cluster of immune pathways including those mediated by T_H_17, T_H_1, and T_H_2 cells (Supplementary Fig. S4). Perturbations in immune responses mediated by T_H_17, T_H_1, and T_H_2 have been noted in SZ patients^56,57^. Additionally, the marker genes of oligodendrocytes and astrocytes showed preferential enrichment in DGN2 (compared to all the other DGNs) (Fig. 3d). Interestingly, T_H_17-mediated immune processes have been known to play critical roles in neuroinflammation and neurodegeneration by regulating the functions of both oligodendrocytes and astrocytes^58–61^. DGN2 also showed the highest/exclusive enrichment (compared to other DGNs) for the pathways and GWAS genes associated with the immune diseases RA and SLE (Supplementary Fig. S4). It was also exclusively enriched for genes associated with deficits in sensorimotor adaptation measured using anti-saccade and prepulse inhibition tasks (Supplementary Fig. S5). Immune deficits have been noted in a SZ mouse model of prenatal immune activation, which is characterized by delayed onset of abnormalities in prepulse inhibition^65^. Additionally, impairments in prepulse inhibition and hippocampal neurogenesis had an early adulthood onset in congenitally immune-deficient mice with severe combined immune deficiency (SCID)^65^. Multiple neurotransmitter systems have been shown to regulate sensorimotor gating^86^. In line with this, we found that DGN2 was clearly distinguished from DGN1 (which showed marked enrichment for dopaminergic marker genes), because of their enrichment among cholinergic, glutamatergic, GABAergic, and serotonergic marker genes (Fig. 3c).

Overall, the observations suggested that the SZ interactome contained genes belonging to the temporal clusters DGN1 and DGN2, underlying different SZ subtypes, as speculated by others^87^.

DGN3 contained genes uniquely found in the PD interactome. This gene cluster was notable for its lack of regional specificities, peak expression in specific developmental stages (10mos, 4 years, 8 years, 11 years, and 40 years), and potential involvement in protein degradation pathways operating in the mitochondria. Further investigations are necessary to characterize this gene cluster. Nevertheless, several factors provide clues to its phenotypic affiliations. The interactome of genes associated with Lewy body dementia (LBD) (Supplementary Data File S6) clustered with DGN3 (Fig. 4a)^46^. The LBD constitute two broad clinical syndromes, namely, dementia with Lewy bodies, and PD-dementia, and psychotic symptoms are often observed^4–6^. Both DGN3 and the LBD interactome showed significant enrichment in post-mortem substantia nigra and prefrontal cortex samples of PD patients and cell lines derived from PARKIN-deficient patients (Supplementary Data File S7).

Altogether, our study provides a bird’s eye view of the processes that may explain the overlaps between PD and SZ. Embedded within the SZ interactome are two distinct groups of genes with different regional and temporal specificities, namely, DGN1 expressed across prenatal stages and later in life (associated with neurodevelopmental processes), and DGN2 with expression from infancy to adulthood (associated with immune-related processes). DGN3 found from the PD interactome is spatially distributed, and spread over time, and is involved in mitochondrial protein degradation pathways. Finally, the prenatally expressed DGN4 contains genes shared between PD and SZ interactomes. We observe that DGN4 overlaps with the interactome of 22q11 deletion syndrome. Future assessments of the genetic architecture of PD and SZ could focus on these four gene clusters.

### Disease development models suggested by the DGNs

Our results hint at two pathways that could contribute to PD and SZ. One, we suggest that there are specific windows of risk, for both PD and SZ, that are exposed by perturbed neurodevelopmental and regeneration-related pathways (as identified by our analysis), over the lifespan^88^. This then allows other processes, such as inflammation, to increase disease risk^89,90^. For example, perturbations in DGN4 could interact with the perturbations in DGN2, giving rise to the inflammatory subtype of SZ. It is important to note that, individual genetic variability, and environmental and lifestyle factors, may influence the proposed causal relationship of disease risk with a specific set of interacting pathways in myriad ways. Moreover, genetic and environmental factors may confer additive, interactive, or protective effects in varying risk periods and modify the disease phenotype.

Two, the DGNs could reflect the multiple facets of these two disease syndromes. Depending on the clustering of disease-associated variants in a specific DGN, a specific disorder subtype could manifest^91,92^, for example, DGN4 with PD/SZ comorbid symptoms, such as in 22q11 deletion syndrome. In the same vein, DGN1 may be more critical to understanding early onset SZ, which often overlaps with neurodevelopmental processes, and DGN2 to the onset of SZ later in life, with inflammatory processes playing an additional role. However, it is important to note that due to the genetic and clinical heterogeneity inherent to these disorders, it is not feasible to exclusively attribute specific disease subtypes to particular sets of disease-associated variants, at this point in time.

### Limitations of the study

Our study has a few limitations (Supplementary Note S8) that should be addressed in future studies. Our analyses should be updated as the genetic architecture of PD and SZ continues to expand. Using DisGeNET – which compiles gene-disease associations from various sources, including genetic associations in model organisms, disease-related differential gene expression, post-translational modifications, and gene-dependent responses to therapeutics – introduces complex factors, beyond disease genetics into our analyses. This warrants caution when interpreting the functional implications of the DGNs. However, the statistically significant replication of DGN3 and DGN4, and the recapitulation, albeit with less significance, of DGN1 and DGN2, using updated PD and SZ interactomes incorporating GWAS and exome-sequencing data, enhances the credibility of our findings. The genetic data from DisGeNET is older as compared to that from recent GWAS and exome-sequencing studies^69–76^, and the corresponding datasets have only a minimal overlap (9 genes for SZ and 12 for PD). This is not surprising as this characteristic has been noted in prior works in SZ where 25 historically considered genes and 77 genes from meta-analysis of GWAS data showed only one gene in common^18,93^. However, the interactome analysis of these two gene sets showed a biological congruence in terms of relevant pathways spanning across the interactomes^25^. Likewise, in this study, the overall interactomic landscape from both sources of data seems to provide similar insights. Our selection of the 100 top-ranking genes – with varying ranges of GDA scores for PD and SZ –could have resulted in an imbalance in the strength of evidence of the two interactomes. However, this may, in part, be a natural consequence of the difference in the research scales of both the disorders (for additional details, see Supplementary Methods).

Furthermore, to enhance the reliability of our findings, which are based on a diverse set of publicly available regional expression, cell-type and pathway datasets, the statistical results need to be replicated with larger datasets as they become available. Lastly, while our study integrates spatiotemporal data, the interactome model remains static, limiting its ability to account for the full complexity of the genetic and environmental events that influence the development of the two disease syndromes.

In summary, we assembled protein interactomes of PD and SZ associated genes, and using spatiotemporal expression data from BrainSpan Atlas and GTEx, conducted clustering and enrichment analyses to identify gene clusters. Our study revealed four possible clusters in space (brain regions) and time (lifespan) that could contribute to the risk and the comorbidity of various symptoms in PD and SZ.

## Methods

### Hierarchical clustering and principal component analysis of disease interactome spatiotemporal expression profiles

The top hundred genes linked to PD (DisGeNET ID: C0030567) and SZ (DisGeNET ID: C0036341) were compiled from the DisGeNET database^34^ (version 7; Supplementary Data File S1) based on their gene-disease association scores (Supplementary Methods). The PPI repositories BioGRID^36^ (version 4.3.194) and HPRD^35^ (version 9) were used to compile the PPIs – specifically, direct interactions (MI:0407) and physical associations (MI:0915) – of (the proteins encoded by) the disease-associated genes (Supplementary Data File S1) and construct the PD and SZ interactomes (Supplementary Methods). We compiled spatiotemporal RNA-sequencing expression data from the BrainSpan Atlas^37^ for all the genes that occur in either of the interactomes. Overall, 4,629 genes were present in PD and SZ interactomes. The expression values of the genes were given in RPKM. Average RPKM values were calculated for multiple brain samples dissected in the same spatiotemporal conditions. Unexpressed genes showing RPKM = 0 were treated as n/a. This yielded a data matrix of genes versus spatiotemporal points, which contained the spatiotemporal profiles of 4,436 out of the 4,629 genes present in the PD and SZ interactomes across 407 spatiotemporal points encompassing 26 brain regions and 10 developmental stages. The RPKM values were log_10_ transformed to reduce the influence of extreme values. Hierarchical clustering was performed on log_10_RPKM values in this matrix using the Morpheus software^94^. Pairwise distances in the data matrix were calculated using Pearson correlation and closely linked clusters were identified using the average linkage method. The average linkage method, also known as Unweighted Pair Group Method with Arithmetic Mean (UPGMA), hierarchically clusters data points by iteratively merging the closest clusters based on their smallest average pairwise distances until all data points form a single cluster. This method was chosen in the study for its ability to produce balanced and elongated clusters by calculating the average pairwise distance between all pairs of data points^95^. In contrast, the single linkage method (that computes the minimum distance between two data points), while effective at identifying compact clusters, may result in chained data points^95^. The complete linkage method (that computes the maximum distance between two data points), while better at capturing outliers, might lead to unbalanced cluster sizes^95^. PCA was carried out on the spatiotemporal expression matrix containing the log_10_RPKM values of 4436 genes in PD and SZ interactomes versus 407 spatiotemporal points (see Supplementary Methods).

### Gene expression enrichment analysis of DGNs

We checked the enrichment of DGNs among genes expressed in specific brain regions compiled from GTEx (version 8)^49^, and the Developmental Transcriptome and LMD Microarray datasets available in the BrainSpan Atlas (see Supplementary Methods)^46^. The gene matrix transpose files prepared from these datasets served as inputs for a gene over-representation analysis based on hypergeometric distribution (see Supplementary Methods).

### Functional enrichment analysis of DGNs

Following the methodology detailed by Li et al.^96^, we decomposed the human interactome into 241 topological modules using the Cytoscape plugin MCODE (see Supplementary Methods)^97^. Pathway (KEGG^39^), differential gene expression datasets, brain cell type and SZ endophenotype enrichments for the various DGNs were computed using WebGestalt (see Supplementary Methods)^53^.

Note that the p-values derived from the enrichment analyses were corrected for multiple hypothesis using the Benjamini-Hochberg (BH) method. In this method, the hypergeometric p-values are sorted from small to large, and multiplied by the total number of tests and then divided by its rank order. A *p*-value < 0.05 after BH correction was considered to be statistically significant. The BH method effectively controls for FDR, which represents the expected proportion of false positives among all positives that reject the null hypothesis. The BH method also produces a stronger correlation between raw and FDR-adjusted p-values, and adjusts well for false negatives in addition to false positives compared to other multiple hypotheses correction methods such as Bonferroni and permutation-based correction methods, indicating its higher reliability^98^. The gene sets used for the various enrichment analyses in this study can be found in Supplementary Data File S8. Genes highly expressed in prenatal structures available in the BrainSpan Atlas were curated from the Harmonizome database^99^ (https://maayanlab.cloud/Harmonizome/) and the genes for pathway enrichment analysis were curated from the KEGG database^39^ (https://www.genome.jp/kegg/pathway.html).

### Supplementary information

Supplementary Information File

Supplementary Data Files

## Data Availability

The PD and SZ-associated genes and interactomes, 22q11 deletion syndrome-associated genes and interactome, and LBD-associated genes and interactome, and the gene sets used for various enrichment analyses in the study have been made available as Supplementary Data Files S1, S5, S6 and S8 respectively.
