# Author Correction: Parkinson’s disease and schizophrenia interactomes contain temporally distinct gene clusters underlying comorbid mechanisms and unique disease processes

**DOI:** 10.1038/s41537-024-00455-3

**Published:** 2024-03-13

**Authors:** Kalyani B. Karunakaran, Sanjeev Jain, Samir K. Brahmachari, N. Balakrishnan, Madhavi K. Ganapathiraju

**Affiliations:** 1 Supercomputer Education and Research Centre, Indian Institute of Science, Bangalore, Karnataka India; 2 Institute for the Advanced Study of Human Biology, Kyoto University, Kyoto, Japan; 3 National Institute of Mental Health and Neuro-Sciences (NIMHANS), Bangalore, Karnataka India; 4 Academy of Scientific and Innovative Research, CSIR-4PI Bangalore, Karnataka India; 5 Department of Computer Science, Carnegie Mellon University Qatar, Doha, Qatar; 6 Department of Biomedical Informatics, School of Medicine, University of Pittsburgh, Pittsburgh, PA USA

**Keywords:** Schizophrenia, Genetics of the nervous system

Correction to: *Schizophrenia* 10.1038/s41537-024-00439-3, published online 27 February 2024

“In the Acknowledgements section, the funding source ‘The Open Access costs for this publication were supported by the Carnegie Mellon University Libraries.’ should have read ‘The Open Access costs for this publication were supported by the Qatar National Library.’ The original article has been corrected.”

