# Supplementary material for: Parkinson’s disease and schizophrenia interactomes contain temporally distinct gene clusters underlying comorbid mechanisms and unique disease processes
Source: Schizophrenia (Heidelb). 2024 Feb 27;10(1):26. doi: 10.1038/s41537-024-00439-3 (PMC10899210; doi:10.1038/s41537-024-00439-3)
Supplement: Supplementary file 1 — Supplementary Information File [file 41537_2024_439_MOESM1_ESM.docx]

## Supplementary Information

Parkinson’s disease and schizophrenia interactomes contain temporally distinct gene clusters underlying comorbid mechanisms and unique disease processes

**Authors:**

Kalyani B. Karunakaran^1,2*^, Sanjeev Jain^3*^, Samir K. Brahmachari^4^, N. Balakrishnan^1^ and Madhavi K. Ganapathiraju^5,6*^

**Affiliations:**

^1^Supercomputer Education and Research Centre, Indian Institute of Science, Bangalore, India

^2^Institute for the Advanced Study of Human Biology, Kyoto University, Kyoto, Japan

^3^National Institute of Mental Health and Neuro-Sciences (NIMHANS), Bangalore, India

^4^Academy of Scientific and Innovative Research, CSIR-4PI, Bangalore, India

^5^Department of Computer Science, Carnegie Mellon University Qatar, Doha, Qatar

^6^Department of Biomedical Informatics, School of Medicine, University of Pittsburgh, Pittsburgh, PA, USA

***Correspondence to:** Kalyani B. Karunakaran ([kalyanithepebble@gmail.com](file:///D:\Users\ekaru\D:\Users\ekaru\Documents\NBK-2020-2022-4\Drug%20contraindications%20in%20comorbidities\Results\Parkinson-Disease-Schizophrenia-Networks-v2\Internal%20Revision\Translational%20Psychiatry\kalyanithepebble@gmail.com)), Sanjeev Jain ([sjain.nimhans@gmail.com](mailto:sjain.nimhans@gmail.com)) and Madhavi K. Ganapathiraju ([madhavi@cs.cmu.edu](mailto:madhavi@cs.cmu.edu))

This file contains:

- Supplementary Methods
- Supplementary Notes S1 to S8
- Supplementary Figures S1 to S10
- Supplementary Tables S1 to S8
- Supplementary References 1 to 38

**Supplementary Methods**

**Compilation of disease associated genes and construction of interactomes**

One hundred top-ranking genes associated with PD (DisGeNET ID: C0030567) and SZ (DisGeNET ID: C0036341), respectively, were compiled from the DisGeNET database^1^ (version 7) (**Supplementary Data File S1**) based on their gene-disease association (GDA) scores. Although the range of the GDA scores across these genes varied for SZ (0.43-0.9) and PD (0.23-0.7), a minimum GDA of 0.01 was chosen to ensure that at least one publication had linked the gene in question with the disease. Note that ‘association’ of a gene with a disease here does not imply causality in most cases, and may only indicate an association with disease susceptibility or an endophenotype.

The GDA score for a gene is computed based on various factors, including the number of supporting publications, the types and number of database sources, and validations in model organisms. Typically, a stringent GDA threshold of ≥ 0.7 is used to select disease-associated genes from the DisGeNET database. However, for PD, only one gene, SNCA, meets this criterion. Setting a lower threshold of GDA ≥ 0.5 would result in selecting only 17 PD-associated genes against 73 SZ-associated genes, causing imbalanced interactome sizes. This imbalance could skew statistical analyses, potentially overestimating the rarity of PD interactome genes and underestimating the rarity of SZ interactome genes in the pool of all interactome genes (as per data from the PPI repositories).

To address this issue, we selected the top 100 genes associated with each disease, ensuring the inclusion of genes with the greatest number of supporting pieces of evidence (GDA ≥ 0.7). However, the caveat of this approach is the inclusion of weaker gene-disease associations in the analyses. Nevertheless, one could argue that this is a reflection of the variations in the evidence base for these diseases. Publications supporting SZ GDAs often stem from large-scale efforts to identify SZ-associated genetic variations in large SZ cohort sizes.^2,3^ These efforts, such as the studies conducted by the SZ Working Group of the Psychiatric Genomics Consortium, are often followed by replication studies in different cohorts, or small-scale targeted investigations. In contrast, research on PD genetics is dispersed across multiple publications that report findings from smaller PD cohorts,^4-8^ limiting replication and the scope of targeted investigations. This difference in the size and nature of the datasets may contribute to the accumulation of more pieces of evidence supporting SZ than PD. This, in turn, is reflected in the ranges of their GDA scores, i.e. 0.43-0.9 for SZ, and 0.23-0.7 for PD. Therefore, our approach of selecting the 100 top-ranking genes, including those that meet the stringent criterion (GDA ≥ 0.7), is an attempt to provide a sufficiently diverse and nuanced view of the interactome landscapes of both the disorders.

PD and SZ interactomes were assembled by extracting the PPIs of the proteins encoded by the disease-associated genes from the PPI repositories BioGRID^9^ (version 4.3.194) and HPRD^10^ (version 9) using BisoGenet (**Supplementary Data File S1**).^11^ The network building options in BisoGenet were: organism - *Homo sapiens*, biorelation type - *protein-protein interaction*, data sources - *BioGRID and HPRD*, method - *input nodes and its neighbors up to a distance of 1*.

**Compilation of LBD and 22q11 deletion associated genes and construction of interactomes**

Twenty-three genes associated with LBD were compiled from the GWAS catalog, and the LBD interactome containing 354 nodes was constructed using BioGRID^9^ and HPRD^10^ data as described before (**Supplementary Data File S6**). Seventy-seven genes differentially expressed in induced pluripotent stem cell-derived neurons of SZ patients with 22q11 deletion were compiled from the Gene Expression Omnibus (GEO) dataset GSE46562,^12^ and the 22q11 deletion interactome containing 1542 nodes was constructed using these genes as input nodes (**Supplementary Data File S5**). Note that the genes were either significantly overexpressed or underexpressed with fold change >1.2 or <$\frac{1}{1.2}$ respectively at p-value < 0.05.

**Principal component analysis of disease interactome spatiotemporal expression profiles**

Following the established approach to reduce the influence of extreme values on the principal components (PCs),^13^ the RPKM values were log_10_-transformed and assembled into a data matrix containing 4,436 genes for PD and SZ interactomes (rows) and 407 brain structures from various developmental phases (columns). Principal component analysis (PCA) was used to transform our original log_10_RPKM variables into uncorrelated variables, and it was implemented using the pca Python package.^14^ The data matrix was pre-processed to retain only rows and columns with less than 70% missing values. The log_10_RPKM values in the matrix were further centered using the unit variance scaling method (row-wise average subtracted from each of the log_10_RPKM values, and the resulting values divided by row-wise standard deviation); this ensures that they assume equal importance while finding the components. The method called singular value decomposition (SVD) with imputation was used to extract principal components.^15^ In this method, missing values are predicted and iteratively filled using neighboring values obtained by SVD until they converge. The number of PCs computed matched the number of column dimensions in the data matrix, representing the number of spatiotemporal conditions in our study. These PCs were ranked in descending order based on the percentage of total variance they explained. We examined the principal component that captured the maximum variance, i.e., PC1, and further extracted the specific spatiotemporal conditions that exhibited the highest loadings on PC1. Component loadings represent correlation coefficients between the original variables (log_10_RPKM values of genes) and the principal components (e.g., PC1, PC2). The squared magnitude of a component loading indicates the percentage of variance explained by a particular original variable, elucidating its contribution to the principal components. Subsequently, we performed PCA on a dataset comprising 4,436 genes and their corresponding log_10_RPKM values in the spatiotemporal conditions exhibiting the strongest influence (i.e., having the highest loadings) on PC1 – namely, 24 pcw A1C, 25 pcw A1C, 37 pcw A1,C and 21 pcw AMY, where A1C is primary auditory cortex and AMY is amygdala – using a web-based tool called ClustVis.^16^

## Gene expression enrichment analysis of DGNs

We checked the enrichment of the DGNs among genes expressed in specific brain regions. Genes having logRPKM > 2 in 26 brain regions that are not housekeeping genes – i.e. 9,638 genes detected in all the tissues with transcripts per million (TPM) ≥ 1, as identified in the Human Protein Atlas^17^ – were compiled from BrainSpan Atlas.^18^ This included amygdaloid complex (3,231 genes), anterior (rostral) cingulate (medial prefrontal) cortex (5,362 genes), caudal ganglionic eminence (3,677 genes), cerebellar cortex (3,542 genes), cerebellum (3,601 genes), dorsal thalamus (3,410 genes), dorsolateral prefrontal cortex (3,473 genes), hippocampus (hippocampal formation) (3,628 genes), inferolateral temporal cortex – area TEv, area 20 (7,000 genes), lateral ganglionic eminence (3,698 genes), medial ganglionic eminence (3,678 genes), mediodorsal nucleus of thalamus (3,442 genes), occipital neocortex (3,790 genes), orbital frontal cortex (3,506 genes), parietal neocortex (3,724 genes), posterior (caudal) superior temporal cortex – area 22c (3,443 genes), posteroventral (inferior) parietal cortex (3,369 genes), primary auditory cortex (core) (3,437 genes), primary motor cortex – area M1, area 4 (3,431 genes), primary motor-sensory cortex (3,628 genes), primary somatosensory cortex – area S1, areas 3,1,2 (3,402 genes), primary visual cortex-striate cortex – area V1/17 (3,436 genes), striatum (3,557 genes), temporal neocortex (3,611 genes), upper (rostral) rhombic lip (3,528 genes) and ventrolateral prefrontal cortex (3,467 genes).

For an independent analysis, we used RNA-seq data from the brains of adult donors available in GTEx.^19^ Genes with high or medium expression (TPM ≥ 9) in 13 brain regions were included, provided that they were not housekeeping genes. A gene matrix transpose (GMT) file was created with amygdala (1,953 genes), anterior cingulate cortex-BA24 (2,269 genes), caudate nucleus (2,229 genes), cerebellar hemisphere (3,978 genes), cerebellum (3,968 genes), cortex (2,706 genes), frontal cortex-BA9 (2,872 genes), hippocampus (1,949 genes), hypothalamus (2,374 genes), nucleus accumbens (2,464 genes), putamen (1,892 genes), spinal cord-cervical c-1 (2,408 genes) and substantia nigra (1,949 genes). We also examined the enrichment of the DGNs for genes showing high expression in each of 516 prenatal brain structures relative to the other structures; these expression profiles of 4 human prenatal samples spanning 4 time points were collected from the Harmonizome database.^20^

The GMT files served as inputs for a gene over-representation analysis based on the hypergeometric distribution. In this method, the p-value is computed from the probability of k successes in s draws (without replacement) from a finite population of size N containing exactly M objects with an interesting feature.

$P\left( X=k \right)=\frac{\left( \begin{aligned} M \\ k \end{aligned} \right)\left( \begin{aligned} N-M \\ s-k \end{aligned} \right)}{\left( \begin{aligned} N \\ s \end{aligned} \right)}$ (1)

N = Total number of genes expressed in any brain region

M = Number of genes expressed in a particular brain region

s = Number of genes in a DGN

k = Number of common genes between K and s (genes in a DGN that are also expressed in a particular brain region)

Cumulative distribution function (CDF) of the hypergeometric distribution was calculated using Microsoft Excel: IF(k>=((s*M)/N),1-HYPGEOM.DIST(k-1,s,M,N,TRUE),HYPGEOM.DIST(k,s,M,N,TRUE)). The formula assesses whether the observed value, k, exceeds or equals the expected threshold value for enrichment calculated as (s*M)/N. If k surpasses this threshold, the cumulative probability is calculated using HYPGEOM.DIST(k-1, s, M, N, TRUE). If k is below the threshold, the cumulative probability is calculated using HYPGEOM.DIST(k, s, M, N, TRUE). The expected threshold for enrichment represents the expected number of successes in a sample of size s drawn from a population of N items, containing M successes. The direction of enrichment is calculated as IF(k=expected,"match",IF(k<expected,"de-enriched","enriched")). This formula categorizes the relationship between the observed value k and the expected value, i.e. (s*M)/N. It indicates whether k matches the expected value, is lower than expected (de-enriched), or is higher than expected (enriched). The fold change of enrichment (or enrichment ratio) is calculated as IF(k<expected,expected/k,k/expected). The formula quantifies the magnitude of change between the observed value k and the expected value. It expresses how many times greater or smaller k is compared to the expected value. If k is less than the expected values, the fold change is calculated as expected/k. If k is greater than the expected value, the fold change is calculated as k/expected.

**Interactome overlap analyses**

The statistical significance of the overlap between PD and SZ interactomes was computed using a hypergeometric test (as explained above) with the following parameters: population size, N =17,992 (total number of genes in BioGRID and HPRD), number of successes in the population, M = 3,200 (number of PD interactome genes), sample size, s = 2,662 (number of SZ interactomes genes), and number of successes, k = 1,232 (number of intersecting genes in PD and SZ interactomes). For comparison of the overlap of PD and SZ interactomes with other disorders, we first selected 100 top-ranking genes associated with Alzheimer’s disease, Celiac disease, Crohn disease and peroxisomal disorders from the DisGeNET database. We then constructed their interactomes using PPIs from BioGRID and HPRD, which resulted in interactomes containing 4,624, 1,905, 2,810 and 632 genes, respectively. We then used hypergeometric tests to derive the p-values denoting the statistical significance of the overlaps of the PD interactome with itself, and SZ, Alzheimer’s disease, Crohn disease, Celiac disease and peroxisomal disorder interactomes, and the overlaps of the SZ interactome with itself, the PD interactome and the interactomes of the four other disorders.

**Functional enrichment analysis of DGNs**

Following the methodology detailed by Li et al.,^21^ we decomposed the human interactome comprising 17,939 proteins and 263,121 interactions – collected from BioGRID^9^ and HPRD^10^ – into 241 topological modules using the Cytoscape plugin MCODE.^22^ The parameters for module identification were as follows: degree cut-off = 2, node score cutoff = 0.1, k-core = 2, and maximum depth = 3. We then examined whether the genes in the DGNs were significantly enriched in any of 241 identified topological modules. The enrichment of the identified modules for specific biological processes (Gene Ontology^23^) was computed using WebGestalt.^24^ The interactome figures were created using Cytoscape.^25^ Pathway (KEGG^26^), differential gene expression (DGE) datasets, brain cell-type, SZ endophenotype, and GWAS trait enrichments for the various DGNs were computed using WebGestalt.^24^ WebGestalt computes the distribution of genes belonging to a particular functional category in the input list and compares it with the background distribution of genes belonging to this functional category among all the genes that belong to any functional category in the database selected by the user. Statistical significance of functional category enrichment is computed using Fisher's exact test and corrected using the Benjamini-Hochberg method for multiple test adjustment. Annotations with FDR-corrected p-value < 0.05 were considered significant. The lists of marker genes that are specifically expressed in neuronal and non-neuronal cell populations of the prefrontal cortex were compiled from a study by Lake et al.,^27^ namely, 79 genes in astrocytes, 157 genes in excitatory cells, 303 genes in inhibitory cells, 44 genes in microglial cells, 103 genes in oligodendrocytes and 52 genes in oligodendrocyte precursor cells (OPCs). Only those genes with log_2_(fold change) ≥ 1 in a given cell type compared to all the other cell types were considered to be cell-specific. The genes associated with 11 neurophysiological and neurocognitive SZ endophenotypes were collected from a study by Greenwood et al.,^28^ namely, abstraction and mental flexibility, anti-saccade task, California verbal learning test, degraded stimulus - continuous performance test, emotional recognition, face memory, letter-number span, prepulse inhibition, sensorimotor dexterity, spatial memory, and spatial processing.

**Supplementary Note S1**

**Genes with causal involvement in PD and SZ found among the 100 top-ranking genes**

Using two expert reviews on PD and SZ genetics, we confirmed that genes causally contributing to the diseases were found among the 100 top-ranking SZ and PD genes. Specifically, the 100 top-ranking SZ genes included eleven genes harbouring structural variations, linked to antipsychotic pharmacology and historically implicated in SZ as positional candidates in linkage studies: *CHRNA7*, *COMT*, *DAOA*, *DISC1*, *DTNBP1*, *HTR2A*, *DRD3*, *NOTCH4*, *NRG1*, *PRODH* and *ZDHHC8*.^29^ However, it is important to note that candidate gene research in SZ has not yielded reproducible results. Specifically, several genes that were consistently replicated across subsequent well-designed GWA studies were not detected by hypothesis-driven candidate gene research.^29^ Additionally, the GWA studies do not support the large effect sizes linked to many historical candidate genes. The 100 top-ranking genes for PD included nine genes identified to harbour rare variants in family-based studies: *ATP13A2*, *GBA*, *LRRK2*, *PARK7*, *PINK1*, *PRKN*, *SNCA*, *TMEM230* and *VPS35*.^30^

**Supplementary Note S2**

**Principal component analysis of spatiotemporal expression profiles of PD and SZ interactomes**

We checked whether the DGNs derived from hierarchical clustering would be recapitulated with principal component analysis (PCA) of the same expression dataset. PC1 and PC2 captured 82.2% and 5.68% of the variance, respectively. In order to identify the brain regions that strongly contributed to the PC that captured the largest percentage of variance,^31^ we inspected their loadings on PC1. Two structures in different developmental stages seemed to be correlated with PC1, namely, primary auditory cortex (A1C) at 24 post-conception weeks (pcw), 25 pcw, and 37 pcw, and amygdala (AMY) at 21 pcw (see **Supplementary Fig. S9** for the component score plot and **Supplementary Fig. S10** for the loadings plot). To delineate the influence of these structures in detail, we performed PCA with 4,436 genes and their corresponding expression values in these 4 spatiotemporal conditions (24 pcw A1C, 25 pcw A1C, 37 pcw A1C and 21 pcw AMY) (**Fig. 2**). This analysis recapitulated the two primary gene clusters derived from hierarchical clustering (**Fig. 2a**) – with PC1’ and PC2’ explaining 89.5% and 6% of the variance – and suggested that regional specificities may underlie their bifurcation (**Fig. 2b**). Quadrant II was found to be enriched for proteins shared between the two interactomes (p-value = 1.45E-07) (**Fig. 2c**). Quadrant III was enriched for proteins uniquely found in the PD interactome (p-value = 6.28E-03) (**Fig. 2d**), and quadrants I (p-value = 7.47E-04) and IV (p-value = 1.46E-03) were enriched for proteins uniquely found in the SZ interactome (**Fig. 2e**). 24 pcw A1C, 25 pcw A1C, 37 pcw A1C, and 21 pcw AMY loaded negatively on PC1’, indicating that the gene expression in these regions may be negatively correlated with the component scores along PC1’. Corroborating this, we found that the genes shared between the two interactomes as well as the genes uniquely found in the PD interactome showed higher expression in these regions compared with the genes uniquely found in the SZ interactome.

**Supplementary Note S3**

**Characterization of DGN3**

DGN3 – enriched for genes uniquely found in the PD interactome – showed the highest enrichment for a cluster of pathways involved in Huntington disease, PD, and the proteasome (**Supplementary Fig. S4**), indicating the exclusive involvement of protein complexes in this DGN-group. Topological module #2 enriched with UCH proteinases (p-value < 1E-12) and modules #8 and #10 enriched with proteins involved in mitochondrial translation were identified (p-value < 1E-12) from DGN3. Together, they captured two different mechanisms underlying early-onset Parkinsonism, namely, PRKN-mediated disruptions in the ubiquitin-proteasome system and PINK1-mediated mitochondrial dysfunctions.^32^ It is notable in this context that DGN3 genes showed high expression at specific temporal points – 10mos, 4yrs, 8yrs, 11yrs, and 40yrs (**Fig. 2a**) in subcortical, frontal cortical, temporal, parietal and sensory-motor regions – possibly reflecting brain-wide disease mechanisms activated during childhood and adolescent phases, resulting in the manifestation of symptoms during middle adulthood. DGN3 clustered with DGN4 based on regional specificities (**Fig. 3a, b**). Higher enrichment for pathway clusters including insulin resistance and cellular senescence (**Supplementary Fig. S4**), both of which have been linked to PD pathogenesis,^33,34^ differentiated this cluster from DGN1 and DGN2. Additionally, module #3 enriched with proteins involved in the transcriptional regulation of white adipocyte differentiation was identified from DGN3 (p-value < 1E-12) and DGN4 (p-value < 1E-13).

**Supplementary Note S4**

**Association of DGN2 with anxiety and depressive disorders**

DGN2 showed exclusive enrichment for a list of 840 genes associated with anxiety disorders (p-value = 2.56E-06) and a list of 1,719 genes associated with depressive disorder (p-value = 4.51E-10), both compiled from the DisGeNET database.^1^ Segregation of DGN2 from DGN1 based on the higher enrichment of the former in limbic structures (as shown in **Fig. 3a, b**) – which play a key role in emotion regulation and show elevated neural activity in clinical anxiety^35^ – further led us to conjecture that DGN2 may be associated with anxiety and depressive disorders. Regions in the limbic circuit were the mostly highly enriched for expression among these anxiety disorder associated genes, namely, putamen, hypothalamus and amygdala (all at p-value = 0.014) as per GTEx, and mediodorsal nucleus of thalamus and striatum (both at p-value = 0.022) as per BrainSpan Atlas. Note that ventrolateral prefrontal cortex, primary motor cortex, orbital frontal cortex, primary auditory cortex and superior temporal cortex were additionally enriched at the same p-value (0.022) as mediodorsal nucleus and striatum. Similarly, depressive disorder associated genes were most enriched for hypothalamus (p-value = 3.03E-05), caudate nucleus (p-value = 1.64E-04), putamen, amygdala and hippocampus (all at p-value = 4.09E-04) as per GTEx, and hippocampus, striatum, amygdala (all at p-value = 1.04E-03) and mediodorsal nucleus of thalamus (p-value = 1.05E-03) as per BrainSpan Atlas. The depressive disorder-associated genes showed statistically significant enrichment (p-value = 3.87E-07) even when they were pruned to contain only 294 expert-curated disease-gene associations with GDA score > 0.2 from the DisGeNET database. However, the list of 51 anxiety disorder-associated genes with a GDA score > 0.2 did not show significant enrichment in DGN2 (p-value = 0.94).

**Supplementary Note S5**

**Association of DGN4 with 22q11 deletion syndrome**

Patients affected with 22q11 deletion syndrome – a genetic disorder arising from microdeletions in the chromosomal region 22q11.2 – are highly susceptible to developing SZ and early-onset PD among a range of other psychiatric and neurodevelopmental disorders.^36^ Human diseases arising from 22q11.2 deletion such as DiGeorge syndrome and velocardiofacial syndrome and animal models of 22q11.2 deletions such as *Df1/+* are useful in investigating the shared etiology of SZ and PD.^36,37^ Therefore, it was interesting to note that the ‘22q11deletion interactome’ constructed from 77 genes differentially expressed in induced pluripotent stem cell-derived neurons of SZ patients with 22q11 deletion (GSE46562^12^) (**Supplementary Data File S5**), showed statistically significant enrichment for DGN4 (p-value = 5.44E-07). The enrichment of this interactome in DGN4 suggested that the genes shared between PD and SZ interactome that are highly expressed in prenatal stages could underlie the comorbidity of psychotic and Parkinsonian symptoms in 22q11 deletion syndrome. Corroborating this further, DGN4 and 22q11 deletion interactome clustered together based on their regional specificities in BrainSpan Atlas (**Fig. 4a**) – i.e. higher enrichment for fetal structures (LGE, MGE, and CGE) – and in GTEx (**Fig. 4b**). Altogether, these results indicate that DGN4 may be involved in both neurodevelopmental processes and disease mechanisms of 22q11 deletion syndrome.

**Supplementary Note S6**

**Recapitulation of the DGNs with additional PPI data**

In our analyses, we compiled the PPIs of PD- and SZ-associated genes from BioGRID and HPRD. To ensure better data coverage, we collected PPIs with medium (0.63) and high (0.7) confidence scores for these genes from the latest release of the HIPPIE database (v2.3).^38^ In HIPPIE, the confidence score of a specific PPI is a weighted sum of the number of studies in which this PPI was detected, the quality and the number of experimental techniques used for detection, and the number of cross-species validations. Augmenting our current PD and SZ interactomes consisting of BioGRID and HPRD PPIs with HIPPIE PPIs expanded the interactome sizes to 4,289 and 3,402, with 1,709 genes in common. Spatiotemporal expression profiles for 94.5% of these genes (5,655 out of 5,982 genes in both interactomes) were available in the BrainSpan Atlas. Hierarchical clustering identified four gene groups with distinct temporal patterns (**Supplementary Fig. S6a**), namely, prenatally-expressed group-I enriched for genes shared between the interactomes, group-II expressed across all the developmental stages and enriched for genes unique to the SZ interactome, group-III expressed in specific postnatal stages and enriched for genes unique to the PD interactome, and postnatally expressed group-IV enriched for genes unique to the SZ interactome (**Supplementary Fig. S6b**). These groups significantly overlapped with DGN4, DGN1, DGN3, and DGN2, respectively (**Supplementary Fig. S6c**). In summary, the expanded interactomes, incorporating HIPPIE PPIs, recapitulated four gene groups with temporal profiles and disease associations corresponding to those of the DGNs. Additionally, they exhibited corresponding overlaps with the DGNs themselves.

**Supplementary Note S7**

**Recapitulation of the DGNs with updated PD and SZ genetic data**

Merging genes from GWAS, exome-sequencing, and family-based studies with our original DisGeNET gene sets yielded 169 PD-associated genes and 219 SZ-associated genes. Upon extracting the PPIs of these genes from BioGRID and HPRD, we obtained PD and SZ interactomes containing 3,871 and 4,079 genes, respectively, with 2,100 genes in common. The spatiotemporal profiles for 96% of these genes (5,614 out of 5,850 genes in both interactomes) were available in the BrainSpan Atlas. Hierarchical clustering of these profiles revealed five gene groups with distinct temporal patterns (**Supplementary Fig. S8a**). Two of these groups demonstrated enrichment for specific gene subsets, namely, the prenatally-expressed group-II for genes shared between the interactomes, and group-IV expressed during specific postnatal stages for genes unique to the PD interactome (**Supplementary Fig. S8b**). Correspondingly, these groups showed overlaps of relatively higher significance for DGN4 and DGN3 compared to other DGNs (**Supplementary Fig. S8b**). Group-I, expressed across various developmental stages, exhibited a p-value just above the 0.05 threshold for genes unique to the SZ interactome (**Supplementary Fig. S8b**) and significantly overlapped with DGN1 (**Supplementary Fig. S8c**). In contrast, while group-III showed a significant overlap with DGN2 (**Supplementary Fig. S8c**), its overlaps with genes unique to the SZ interactome was non-significant (**Supplementary Fig. S8b**). In summary, the updated interactomes, integrating the latest genetic findings of PD and SZ, successfully replicated gene groups that closely matched the temporal profiles and disease associations of DGN2 and DGN3 with high significance, and those of DGN1 with modest significance. The limited or lack of significance of SZ-associated gene clusters could be attributed to the wide-ranging temporal contexts in which genes from SZ GWAs and exome-sequencing studies are active, despite their collective involvement in neuronal function.^2,3^ Further focused investigations are necessary to elucidate these contexts.

**Supplementary Note S8**

**Limitations of the study**

Our study has several limitations. First, we considered only the 100 top-ranking genes associated with PD and SZ out of ~2,000 and ~2,800 genes linked to each of the two disorders, including ~700 and ~1,300 genes supported by genetic variant evidence alone. A more comprehensive analysis with the gene datasets included in their totality, or subject to a threshold criterion other than gene-disease association scores, will provide better insights into PD and SZ biology. Additionally, each of the two disease interactomes contained around 3000 genes, possibly due to the size restriction on the PD and SZ gene lists used to seed the interactomes and comparable topological characteristics of the PD and SZ in the human interactome. Systematic investigations after assembling interactomes based on a more comprehensive disease gene list are necessary to clarify these points.

Second, the Benjamini-Hochberg (BH) method for multiple hypothesis correction was applied to compute the p-values of enrichment of DGN1 and DGN2 for the three subsets of the PD and SZ interactomes, i.e., genes (i) shared between PD and SZ interactomes, (ii) uniquely found in the PD interactome and (iii) uniquely found in the SZ interactome. DGN1 and DGN2 showed enrichment for genes uniquely found in the SZ interactome, albeit with modest p-values of 0.041 and 0.018, respectively. This result leads us to two conclusions: (i) DGN1 and DGN2 are likely to contain genes uniquely found in the SZ interactome compared to the expected number, based on the frequency of unique SZ interactome genes in the human interactome, i.e., this observation is statistically significant at the set threshold of p-value < 0.05, and (ii) the difference in the p-values for DGN1 and DGN-group, i.e., 0.041 and 0.018, could arise from the difference in their numerical overlaps with the set of unique SZ interactome genes, i.e., 313 and 355. Replication with more comprehensive gene sets and other statistical tests is necessary to provide an unequivocal interpretation of these results. The enrichment of DGN2 for oligodendrocyte markers barely crossed the 0.05 threshold after correction using the BH method. The parameters of the datasets used for the hypergeometric test, such as the small number of oligodendrocyte marker genes and the consequent low numerical overlap with DGN2, could be responsible for this modest p-value. Although this enrichment is notable since none of the other cell types showed enrichment for DGN2, it should be verified using more comprehensive marker gene sets.


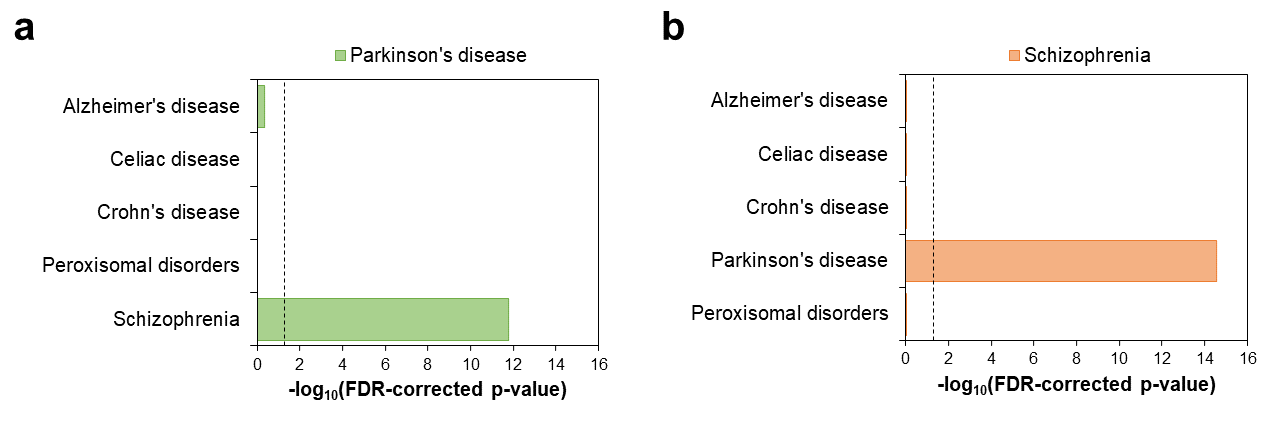
**Supplementary Figure S1: Overlap of PD and SZ interactomes with other disorder interactomes.** Hypergeometric test was used to assess the significance of the overlap of **a** the PD interactome and **b** the SZ interactome with the interactomes of four other disorders, namely, Alzheimer’s disease, celiac disease, Crohn’s disease and peroxisomal disorders. The level of statistical significance has been denoted by -log_10_(FDR-corrected p-value) obtained after multiple test adjustment using the Benjamini-Hochberg method. The dashed black line indicates the cut-off value for –log_10_(p-value) after correction for multiple hypotheses (p-value < 0.05, i.e., log_10_(p-value) > 1.30103).

**
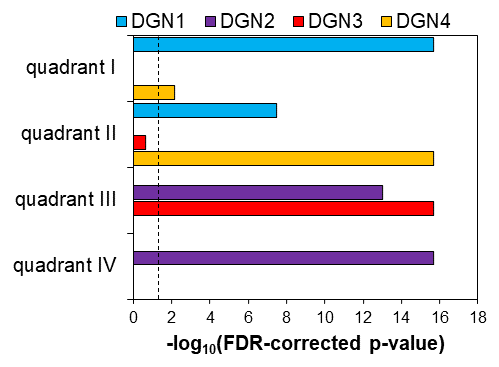
Supplementary Figure S2: Enrichment of disorder interactome genes in corresponding DGNs and PCA quadrants.** Hypergeometric tests were used to assess the significance of the overlap of DGNs 1-4 with PCA quadrants I-IV. The level of statistical significance has been denoted by -log_10_(FDR-corrected p-value) obtained after multiple test adjustment using the Benjamini-Hochberg method. The dashed black line indicates the cut-off value for –log_10_(p-value) after correction for multiple hypotheses (p-value < 0.05, i.e., log_10_(p-value) > 1.30103).


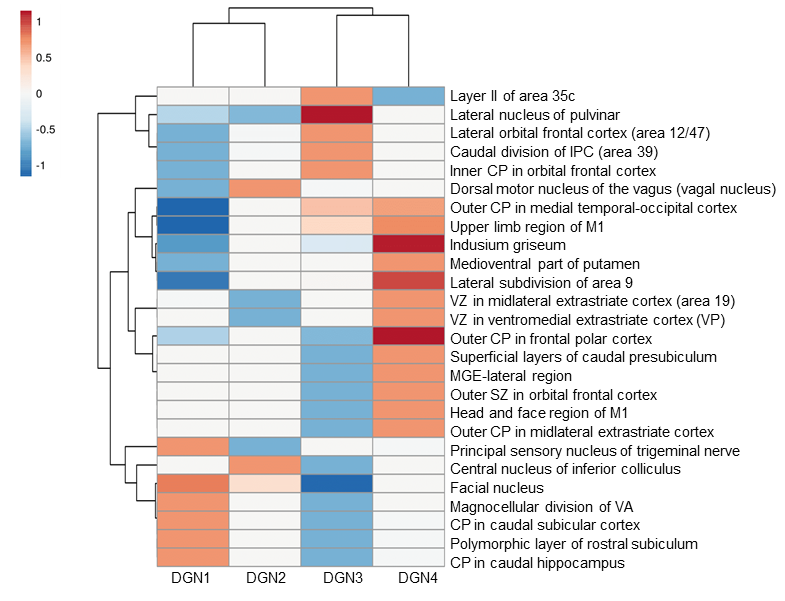
**Supplementary Figure S3: Enrichment patterns of DGNs in 516 prenatal brain structures from BrainSpan Atlas.** The enrichment of the genes belonging to DGNs among those highly expressed in each of 516 prenatal brain structures relative to the other brain structures available in BrainSpan Atlas was checked. The statistical significance of each region-wise enrichment was computed as a p-value. All p-values were transformed to –log_10_p-values, and then assembled into a data matrix containing brain regions as rows and the DGNs as columns. Negative log transformation simplifies the scale, revealing patterns and significant results more visibly, with higher –log10 values signifying smaller p-values. Variations in region-wise enrichment are represented in the form of heat maps. Specifically, z-scores computed based on the inverse normal transformation of –log_10_ transformed p-values are shown. Clustering was performed using the hierarchical clustering method with average linkage. The dendrograms were derived from the clustering analysis based on the computation of Pearson correlation coefficients between the data points. The clustered heat map was created using the ClustVis software. Two main DGN-group clusters were observed: the first cluster included DGN1 and DGN2, and the second cluster included DGN3 and DGN4. Note that this analysis was performed using data collected from a single source (Prenatal LMD Microarray, BrainSpan Atlas).

**
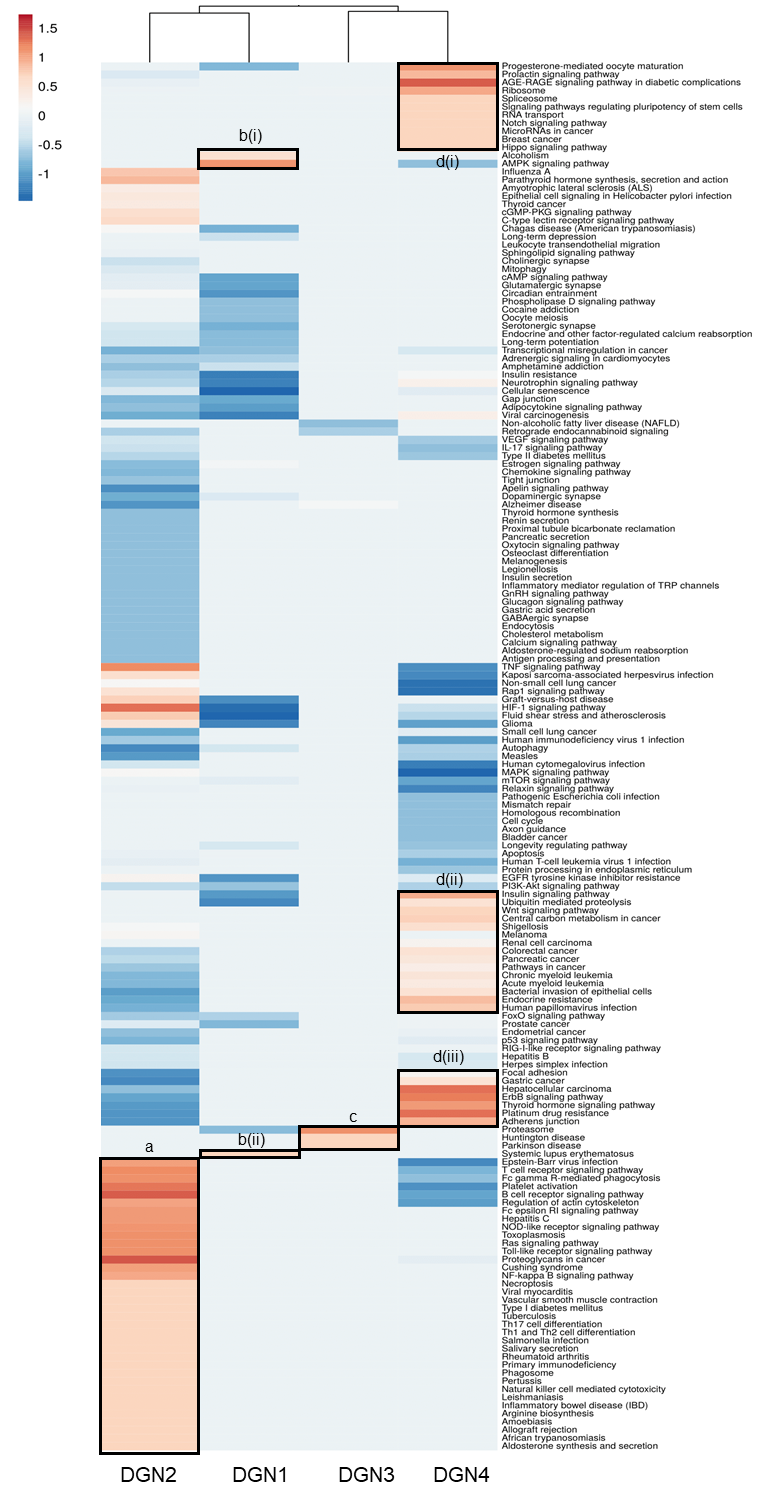
**

**Supplementary Figure S4: Enrichment patterns of DGNs in KEGG pathways.** The enrichment of the genes belonging to DGNs in various pathways was checked using data from KEGG. The statistical significance of pathway enrichment was computed as p-values. These values were transformed to –log_10_P values, and then assembled into a data matrix containing pathways as rows and the DGNs as columns. Negative log transformation simplifies the scale, revealing patterns and significant results more visibly, with higher –log10 values signifying smaller p-values. Variations in pathway enrichment are represented in the form of heat maps. Specifically, z-scores computed based on the inverse normal transformation of –log_10_ transformed p-values are shown. Clustering was performed using the hierarchical clustering method with average linkage. The dendrograms were derived from the clustering analysis based on the computation of Pearson correlation coefficients between the data points. The clustered heat map was created using the ClustVis software. The cluster of immune-related pathways showing high enrichment in DGN2 is marked as ‘a’. So were the clusters showing high enrichment in DGN1 (bi-ii), DGN3 (c) and DGN4 (di-iii). Note that this analysis was performed using data collected from a single source (KEGG database).


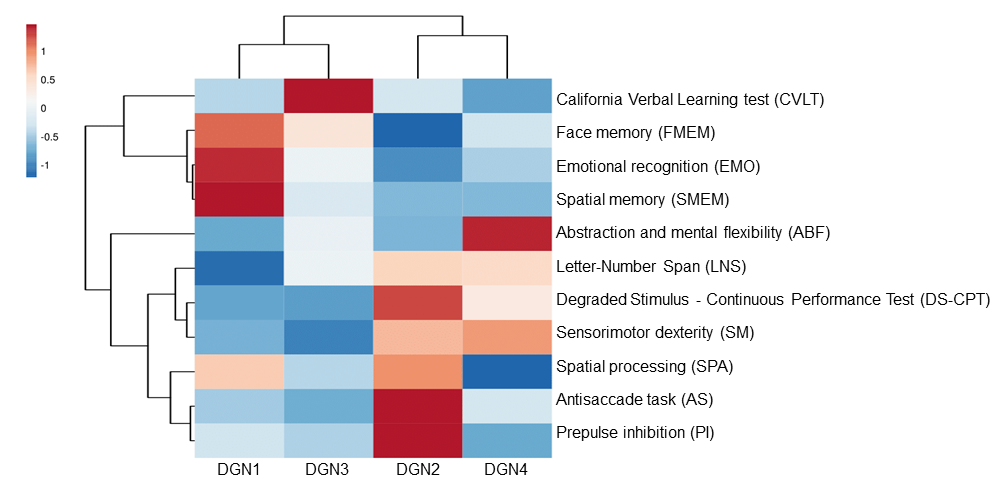
**Supplementary Figure S5: Enrichment of DGNs among genes associated with SZ endophenotypes.** The enrichment of the genes belonging to DGNs among genes associated with 11 SZ endophenotypes compiled from a study by Greenwood et al.^28^ was checked. The statistical significance of endophenotype enrichment was computed as p-values. These values were transformed to –log_10_P values, and then assembled into a data matrix containing brain regions as rows and the DGNs as columns. Negative log transformation simplifies the scale, revealing patterns and significant results more visibly, with higher –log10 values signifying smaller p-values. Variations in endophenotype-wise enrichment have been represented in the form of heat maps. Specifically, normalized z-scores computed based on the –log_10_ transformed p-values are shown. Clustering was performed using the hierarchical clustering method with average linkage. The dendrograms were derived from the clustering analysis based on the computation of Pearson correlation coefficients between the data points. The clustered heat map was created using the Clustvis software. Two main DGN-group clusters were observed: the first cluster included DGN1 and DGN3, and the second cluster included DGN2 and DGN4. Note that this analysis was performed using data compiled from multiple independent studies by Greenwood et al. in a separate study.^28^

**
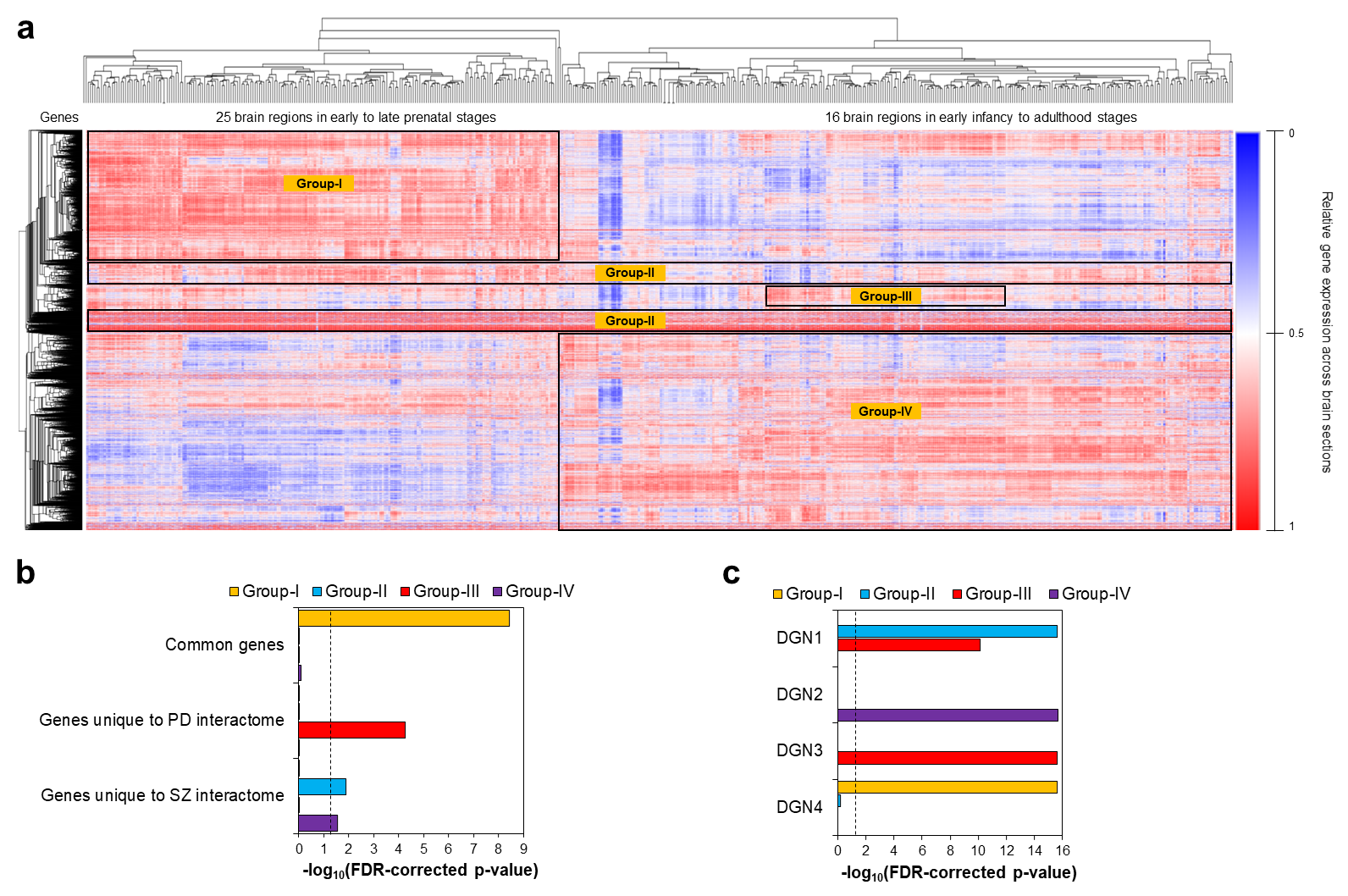
Supplementary Figure S6: Temporal expression patterns of the genes in expanded PD and SZ interactomes incorporating PPIs from the HIPPIE database.** **a** The figure shows the two spatiotemporal clusters – corresponding to 25 brain regions in early prenatal to late prenatal stages (left) and 16 brain regions in early infancy to adulthood stages (right) – on the horizontal axis. Two main gene clusters can be seen on the vertical axis, which can be further subdivided into four sub-clusters (groups 1-IV) showing distinct temporal profiles and preferential enrichment of proteins uniquely found in the PD/SZ interactomes or shared between both the interactomes. Note that these PD and SZ interactomes contained PPIs from three PPI repositories, namely, BioGRID, HPRD and HiPPIE. The expression levels of 5,655 out of the 5,982 genes found in the expanded PD and SZ interactomes were hierarchically clustered across 407 spatiotemporal points. Clustering was performed on log-transformed RPKM (i.e. Reads Per Kilobase per Million mapped reads) values using the hierarchical clustering method with average linkage. The dendrograms were derived from the clustering analysis based on the computation of Pearson correlation coefficients between the data points. The clustered heat map was created using the Morpheus software. Hypergeometric tests were used to assess the significance of the overlap of groups I-IV in **b** different subsets of the expanded PD and SZ interactomes and **c** DGNs (that were derived from the clustering of PD and SZ interactomes without HIPPIE PPIs, and reported in Fig. 2a). The level of statistical significance has been denoted by -log_10_(FDR-corrected p-value) obtained after multiple test adjustment using the Benjamini-Hochberg method. The dashed black line indicates the cut-off value for –log_10_(p-value) after correction for multiple hypotheses (p-value < 0.05, i.e., log_10_(p-value) > 1.30103).

**
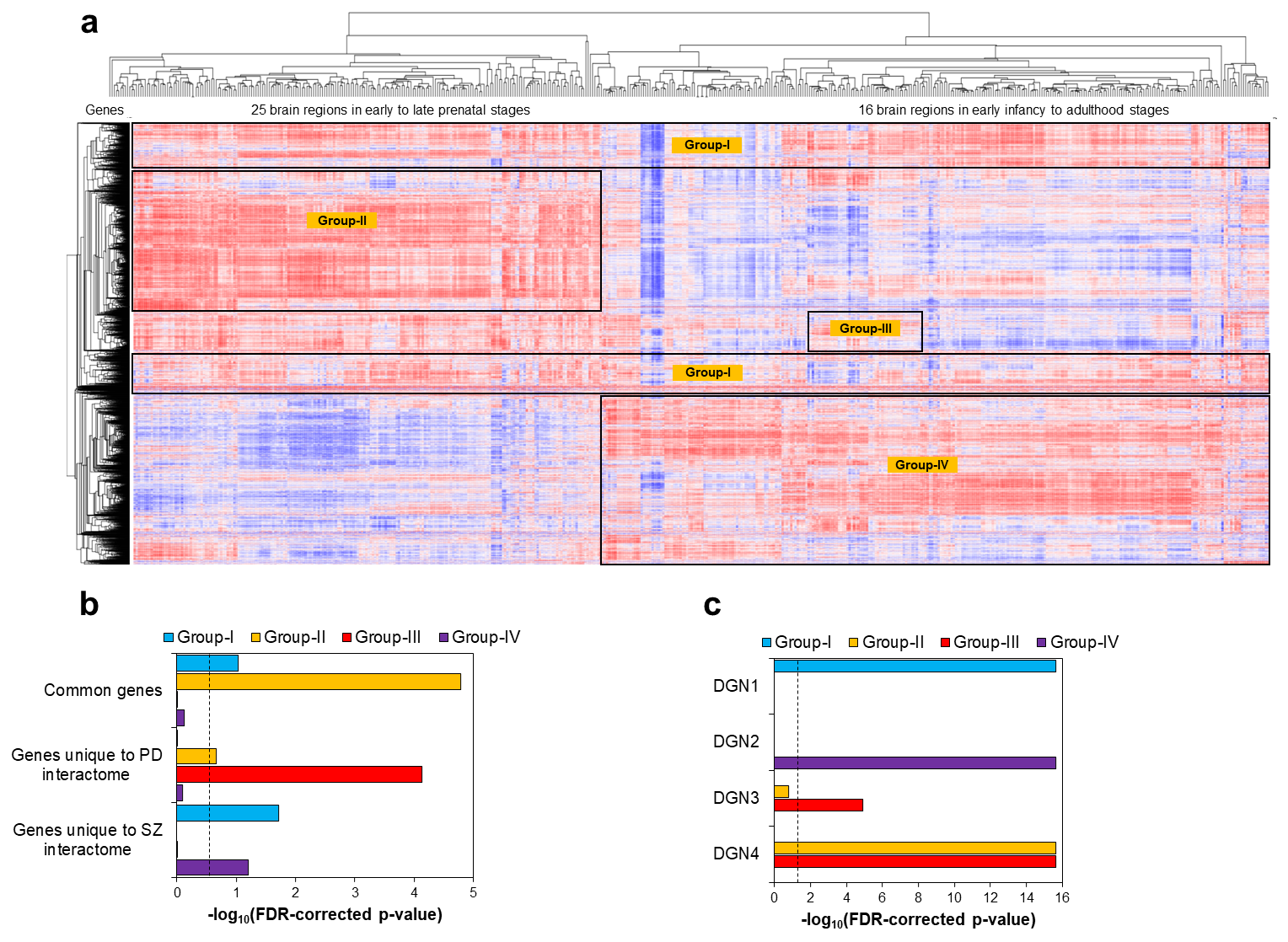
Supplementary Fig. S7: Temporal expression patterns of brain-expressed genes in PD and SZ interactomes.** **a** The figure shows the two spatiotemporal clusters – corresponding to 25 brain regions in early prenatal to late prenatal stages (left) and 16 brain regions in early infancy to adulthood stages (right) – on the horizontal axis. Two main gene clusters can be seen on the vertical axis, which can be further subdivided into four sub-clusters (groups 1-IV) showing distinct temporal profiles and preferential enrichment of proteins uniquely found in the PD/SZ interactomes or shared between both the interactomes. Note that these PD and SZ interactomes contained 3,970 genes that were expressed in the brain (out of a total of 4,629 genes present in both the interactomes), as per data from the Human Protein Atlas. The expression levels of these 3,970 genes expressed in the brain were hierarchically clustered across 407 spatiotemporal points. Clustering was performed on log-transformed RPKM (i.e. Reads Per Kilobase per Million mapped reads) values using the hierarchical clustering method with average linkage. The dendrograms were derived from the clustering analysis based on the computation of Pearson correlation coefficients between the data points. The clustered heat map was created using the Morpheus software. Hypergeometric tests were used to assess the significance of the overlap of groups I-IV in **b** different subsets of the brain-expressed PD and SZ interactomes and **c** DGNs (that were derived from the clustering of PD and SZ interactomes unfiltered for tissue-specificity, and reported in Fig. 2a). The level of statistical significance has been denoted by -log_10_(FDR-corrected p-value) obtained after multiple test adjustment using the Benjamini-Hochberg method. The dashed black line indicates the cut-off value for –log_10_(p-value) after correction for multiple hypotheses (p-value < 0.05, i.e., log_10_(p-value) > 1.30103).

**
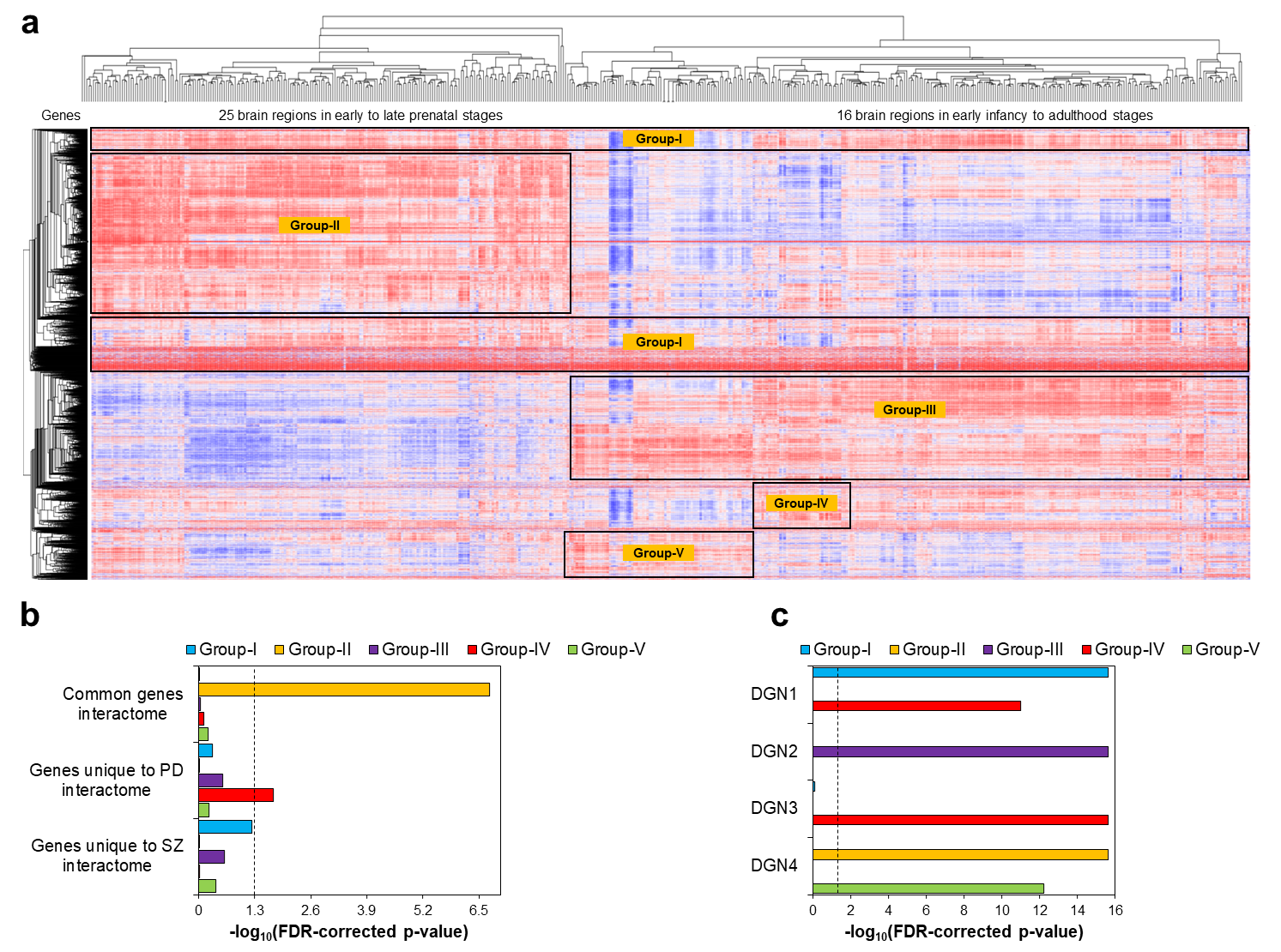
Supplementary Fig. S8: Temporal expression patterns of the genes in updated PD and SZ interactomes incorporating data from recent GWA and exome-sequencing studies.** **a** The figure shows the two spatiotemporal clusters – corresponding to 25 brain regions in early prenatal to late prenatal stages (left) and 16 brain regions in early infancy to adulthood stages (right) – on the horizontal axis. Two main gene clusters can be seen on the vertical axis, which can be further subdivided into four sub-clusters (groups 1-V) showing distinct temporal profiles and preferential enrichment of proteins uniquely found in the PD/SZ interactomes or shared between both the interactomes. Note that these PD and SZ interactomes contained PPIs of the genes identified in eight recent GWA and exome-sequencing studies of PD and SZ; the PPIs were extracted from BioGRID and HPRD. The expression levels of 5,614 out of the 5,850 genes found in both the updated interactomes were hierarchically clustered across 407 spatiotemporal points. Clustering was performed on log-transformed RPKM (i.e. Reads Per Kilobase per Million mapped reads) values using the hierarchical clustering method with average linkage. The dendrograms were derived from the clustering analysis based on the computation of Pearson correlation coefficients between the data points. The clustered heat map was created using the Morpheus software. Hypergeometric tests were used to assess the significance of the overlap of groups I-V in **b** different subsets of the updated PD and SZ interactomes and **c** DGNs (that were derived from the clustering of PD and SZ interactomes constructed based on DisGeNET gene-disease association data, and reported in Fig. 2a). The level of statistical significance has been denoted by -log_10_(FDR-corrected p-value) obtained after multiple test adjustment using the Benjamini-Hochberg method. The dashed black line indicates the cut-off value for –log_10_(p-value) after correction for multiple hypotheses (p-value < 0.05, i.e., log_10_(p-value) > 1.30103).


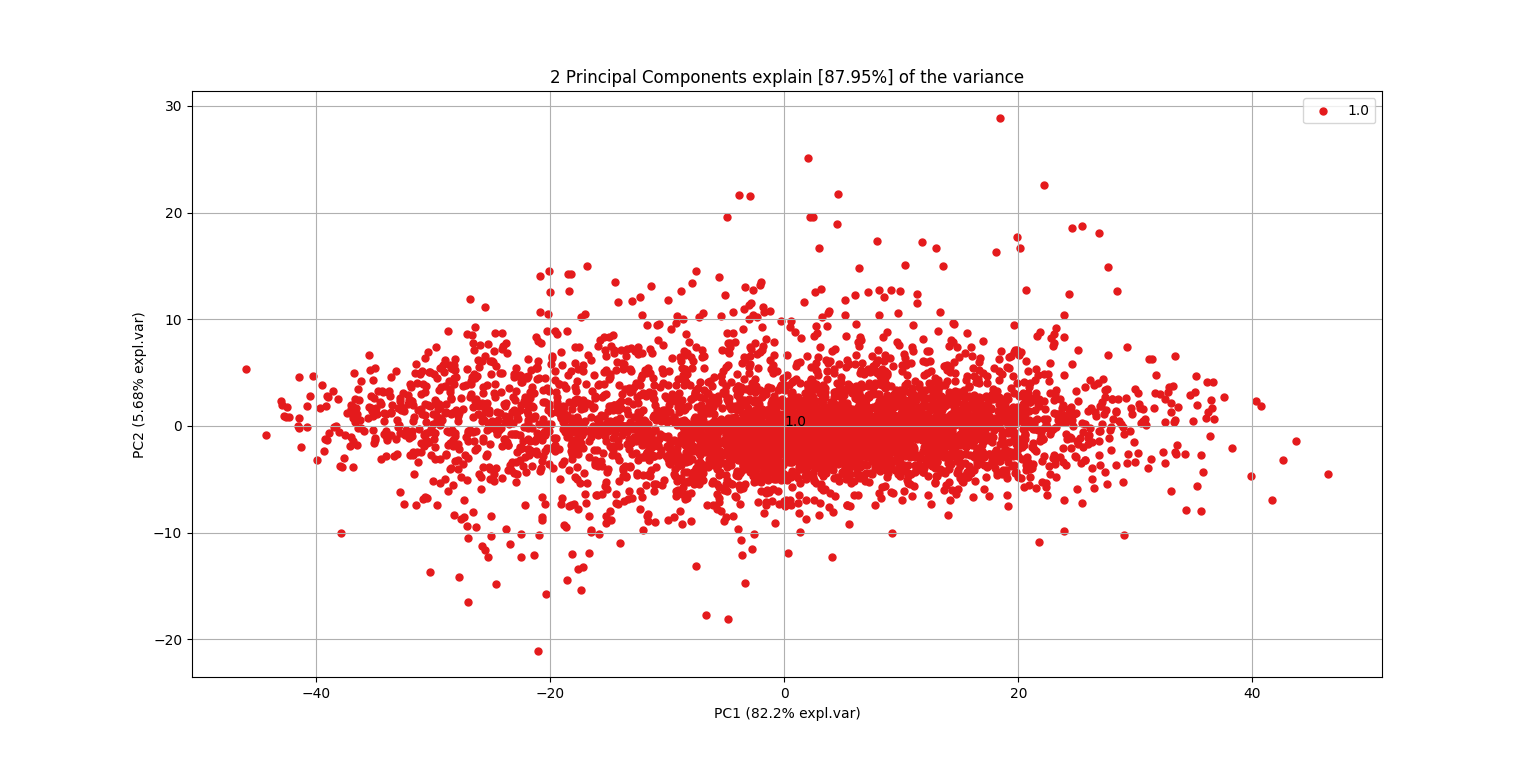
**Supplementary Figure S9: Principal component analysis of the genes present in PD and SCZ networks based on their expression in developing and adult brain structures.** Principal component analysis (PCA) was performed with the expression profiles of the genes in PD and SCZ networks in 407 brain regions. A matrix with 4,436 genes (rows) and 407 brain regions (columns) was constructed out of log-transformed RPKM values. Unit variance scaling was applied across this matrix. Singular value decomposition (SVD) with imputation was used to extract principal components (PCs). Component scores corresponding to PC1 and PC2 explaining 82.2% and 5.7% of the total variance were plotted along X and Y axes respectively.


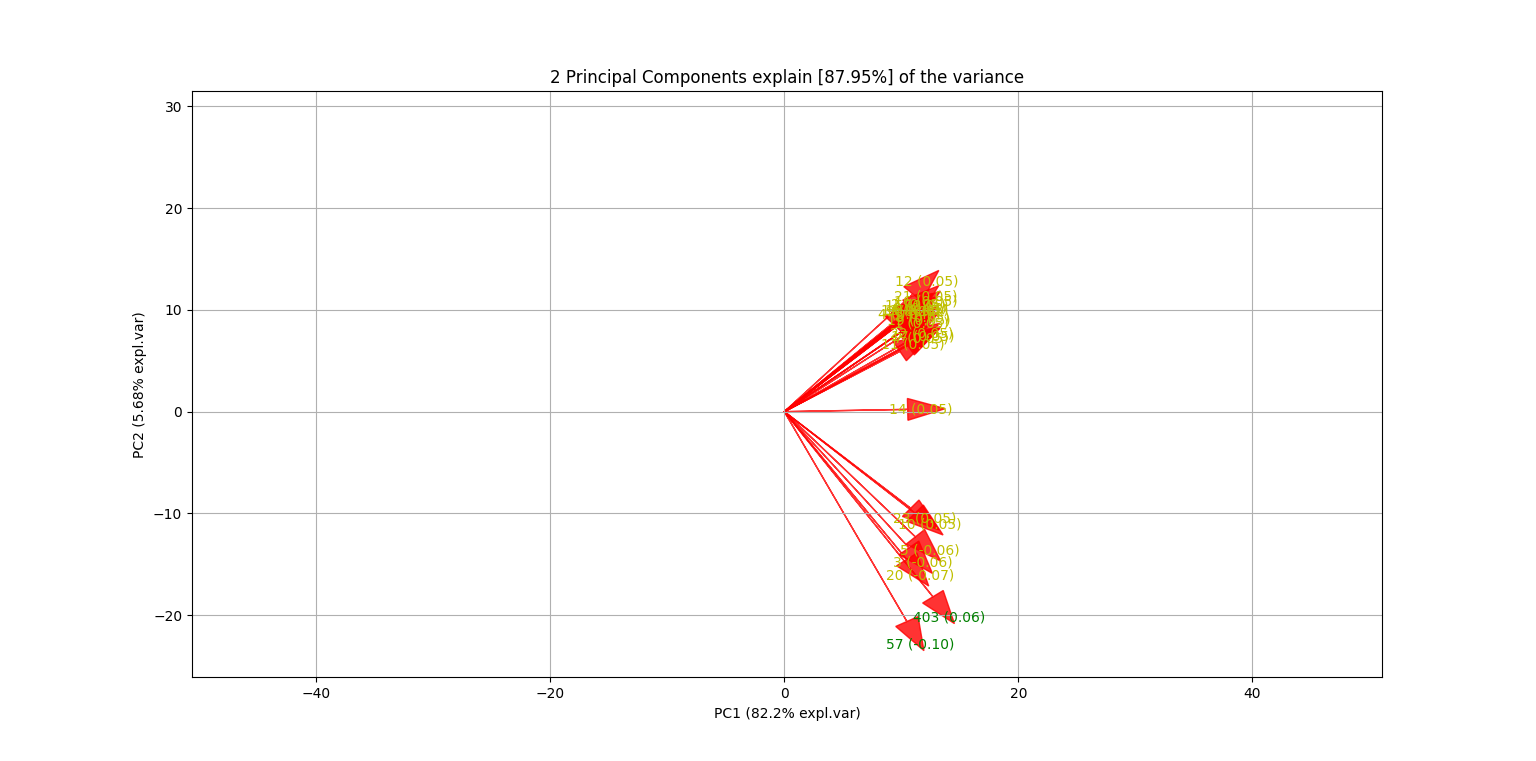
**Supplementary Figure S10: Brain structures potentially influencing the distinct sets of genes present in PD and SCZ networks.** The component loadings of the brain structures (indicated with numbers) are shown as vectors. The brain structures numbered 10, 11, 14 and 23 corresponding to A1C-24pcw, A1C-25pcw, A1C-37pcw and AMY-21pcw seemed to have high loadings on PC1.

**Supplementary Table S1: Cell type specificities of DGN1-DGN4.** The -log_10_p-values of enrichment of the DGNs in six cell types. Note that this is the data underlying Fig. 3d.

| Cell type | DGN1 | DGN2 | DGN4 |
| --- | --- | --- | --- |
| Astrocytes | 0 | 1.15922 | 0.01283 |
| Excitatory cells | 2.10547 | 6.5E-05 | 0.01283 |
| Inhibitory cells | 0 | 6.5E-05 | 0.01283 |
| Microglia | 0 | 6.5E-05 | 0.73634 |
| Oligodendrocytes | 0 | 1.3063 | 0.01283 |
| OPCs | 0 | 6.5E-05 | 1.00955 |

**Supplementary Table S2: Pathway specificities of DGN1-DGN4.** The -log_10_p-values of enrichment of the DGNs in KEGG pathways. Note that this is the data underlying Supplementary Fig. S4.

| Pathway | DGN1 | DGN2 | DGN3 | DGN4 |
| --- | --- | --- | --- | --- |
| Acute myeloid leukemia |  | 3.39563 |  | 4.44275 |
| Adipocytokine signaling pathway | 1.71361 | 1.93307 |  |  |
| Adrenergic signaling in cardiomyocytes | 3.85994 | 3.87452 |  |  |
| African trypanosomiasis |  | 1.74833 |  |  |
| AGE-RAGE signaling pathway in diabetic complications |  | 3.90557 |  | 4.44275 |
| Alcoholism | 12.0434 |  |  |  |
| Aldosterone synthesis and secretion |  | 4.59163 |  |  |
| Aldosterone-regulated sodium reabsorption |  | 3.19359 |  |  |
| Allograft rejection |  | 4.97957 |  |  |
| Alzheimer disease |  | 1.93307 | 3.58886 |  |
| Amoebiasis |  | 2.90079 |  |  |
| Amphetamine addiction | 4.01507 | 3.26021 |  |  |
| AMPK signaling pathway | 4.12015 |  |  | 2.27104 |
| Amyotrophic lateral sclerosis (ALS) |  | 5.32873 |  |  |
| Antigen processing and presentation |  | 5.60744 |  |  |
| Apelin signaling pathway |  | 2.49053 |  |  |
| Apoptosis |  | 4.72349 |  | 3.59459 |
| Arginine biosynthesis |  | 3.12802 |  |  |
| Ascorbate and aldarate metabolism |  |  |  |  |
| Autophagy | 2.65642 | 1.89262 |  | 2.46369 |
| B cell receptor signaling pathway |  | 6.12446 |  | 1.53507 |
| Bacterial invasion of epithelial cells |  | 1.37939 |  | 2.75989 |
| Bile secretion |  |  |  |  |
| Calcium signaling pathway |  | 7.40117 |  |  |
| cAMP signaling pathway | 2.01428 | 6.06065 |  |  |
| Cardiac muscle contraction |  |  |  |  |
| Cellular senescence | 1.40008 | 4.21668 |  | 4.46403 |
| Central carbon metabolism in cancer |  | 2.56584 |  | 2.6905 |
| cGMP-PKG signaling pathway |  | 6.06065 |  |  |
| Chagas disease (American trypanosomiasis) | 1.87601 | 2.97919 |  |  |
| Chemical carcinogenesis |  |  |  |  |
| Chemokine signaling pathway |  | 2.20016 |  |  |
| Cholesterol metabolism |  | 1.72585 |  |  |
| Choline metabolism in cancer |  |  |  |  |
| Cholinergic synapse |  | 3.24156 |  |  |
| Chronic myeloid leukemia |  | 2.34873 |  | 8.1446 |
| Circadian entrainment | 1.41179 | 5.08054 |  |  |
| Circadian rhythm |  |  |  |  |
| Citrate cycle (TCA cycle) |  |  |  |  |
| Cocaine addiction | 2.7505 |  |  |  |
| Colorectal cancer |  | 3.43914 |  | 6.19229 |
| C-type lectin receptor signaling pathway |  | 5.46629 |  |  |
| Cushing syndrome |  | 2.1944 |  |  |
| Dopaminergic synapse | 7.30029 | 3.79118 |  |  |
| Drug metabolism |  |  |  |  |
| EGFR tyrosine kinase inhibitor resistance | 2.01428 | 4.37103 |  | 3.59459 |
| Endocrine and other factor-regulated calcium reabsorption | 2.43344 | 2.8619 |  |  |
| Endocrine resistance |  | 1.44695 |  | 7.47281 |
| Endocytosis |  | 4.05154 |  |  |
| Endometrial cancer |  | 2.11389 |  | 3.29973 |
| Epithelial cell signaling in Helicobacter pylori infection |  | 1.98295 |  |  |
| Epstein-Barr virus infection |  | 8.79011 |  | 4.63451 |
| ErbB signaling pathway |  | 3.48598 |  | 6.89026 |
| Estrogen signaling pathway | 2.65642 | 1.61735 |  |  |
| Fc epsilon RI signaling pathway |  | 3.26021 |  |  |
| Fc gamma R-mediated phagocytosis |  | 4.97957 |  | 2.00953 |
| Fluid shear stress and atherosclerosis | 1.66224 | 4.97957 |  | 3.0889 |
| FoxO signaling pathway | 3.18412 | 3.0341 |  | 4.23627 |
| GABAergic synapse |  | 2.92439 |  |  |
| Gap junction | 1.37282 | 1.49154 |  |  |
| Gastric acid secretion |  | 2.40167 |  |  |
| Glioma | 1.63151 | 3.49451 |  | 1.8806 |
| Glucagon signaling pathway |  | 1.56678 |  |  |
| Glutamatergic synapse | 1.47821 | 5.22813 |  |  |
| GnRH signaling pathway |  | 3.87104 |  |  |
| Graft-versus-host disease | 1.38601 | 4.58284 |  |  |
| Hepatitis B |  | 4.68776 |  | 4.85239 |
| Hepatitis C |  | 3.42073 |  |  |
| Hepatocellular carcinoma |  | 2.59498 |  | 8.76191 |
| Herpes simplex infection |  | 2.90079 |  | 3.07741 |
| HIF-1 signaling pathway | 1.62737 | 5.76655 |  | 3.14097 |
| Human cytomegalovirus infection |  | 5.58263 |  | 3.1268 |
| Human immunodeficiency virus 1 infection |  | 5.08054 |  | 4.054 |
| Human papillomavirus infection |  | 1.76942 |  | 5.48726 |
| Human T-cell leukemia virus 1 infection |  | 4.97883 |  | 3.62933 |
| Huntington disease |  |  | 5.7042 |  |
| IL-17 signaling pathway |  | 1.61735 |  | 1.30172 |
| Inflammatory bowel disease (IBD) |  | 3.45565 |  |  |
| Inflammatory mediator regulation of TRP channels |  | 3.90557 |  |  |
| Influenza A |  | 6.0223 |  |  |
| Insulin resistance | 1.40008 | 2.04212 |  | 2.69911 |
| Insulin secretion |  | 3.09164 |  |  |
| Insulin signaling pathway | 2.33233 |  |  | 6.70026 |
| Kaposi sarcoma-associated herpesvirus infection |  | 9.33659 |  | 2.00953 |
| Legionellosis |  | 1.86413 |  |  |
| Leishmaniasis |  | 6.40841 |  |  |
| Leukocyte transendothelial migration |  |  |  |  |
| Longevity regulating pathway | 3.18412 |  |  | 2.79825 |
| Long-term depression | 2.59014 |  |  |  |
| Long-term potentiation | 3.11522 | 3.79881 |  |  |
| MAPK signaling pathway |  | 5.97829 |  | 2.15099 |
| Measles |  | 1.50015 |  | 1.99076 |
| Melanogenesis |  | 1.63696 |  |  |
| Melanoma |  | 2.58124 |  |  |
| Metabolism of xenobiotics by cytochrome P450 |  |  |  |  |
| Mitophagy |  | 1.74932 |  |  |
| Morphine addiction |  |  |  |  |
| Natural killer cell mediated cytotoxicity |  | 6.8253 |  |  |
| Necroptosis |  | 3.43914 |  |  |
| Neuroactive ligand-receptor interaction |  |  |  |  |
| Neurotrophin signaling pathway | 1.37175 | 3.26021 |  | 5.23636 |
| NF-kappa B signaling pathway |  | 7.59062 |  |  |
| Nicotine addiction |  |  |  |  |
| NOD-like receptor signaling pathway |  | 5.39859 |  |  |
| Non-alcoholic fatty liver disease (NAFLD) |  |  | 3.67662 |  |
| Non-small cell lung cancer |  | 3.87104 |  | 2.61257 |
| Oocyte meiosis | 3.18412 |  |  |  |
| Osteoclast differentiation |  | 7.02359 |  |  |
| Oxytocin signaling pathway |  | 5.91219 |  |  |
| p53 signaling pathway |  | 1.45067 |  | 2.15099 |
| Pancreatic cancer |  | 3.70192 |  | 5.92843 |
| Pancreatic secretion |  | 1.51192 |  |  |
| Parathyroid hormone synthesis, secretion and action |  | 3.90557 |  |  |
| Parkinson disease |  |  | 4.48054 |  |
| Pathways in cancer |  | 7.02359 |  | 10.0594 |
| Pentose and glucuronate interconversions |  |  |  |  |
| Pertussis |  | 2.34873 |  |  |
| Phagosome |  | 3.87104 |  |  |
| Phenylalanine metabolism |  |  |  |  |
| Phospholipase D signaling pathway | 1.46886 |  |  |  |
| PI3K-Akt signaling pathway | 2.01428 | 2.25342 |  | 2.13795 |
| Platelet activation |  | 2.73535 |  | 1.32132 |
| Platinum drug resistance |  | 1.74932 |  | 5.14348 |
| Porphyrin and chlorophyll metabolism |  |  |  |  |
| Primary immunodeficiency |  | 2.09568 |  |  |
| Prion diseases |  |  |  |  |
| Prolactin signaling pathway |  | 2.69953 |  | 3.53042 |
| Prostate cancer | 1.71361 | 2.85759 |  | 3.35033 |
| Protein processing in endoplasmic reticulum |  |  |  | 2.11268 |
| Proteoglycans in cancer |  | 7.81185 |  | 5.66048 |
| Proximal tubule bicarbonate reclamation |  | 2.86261 |  |  |
| Pyruvate metabolism |  |  |  |  |
| Rap1 signaling pathway |  | 2.22345 |  | 1.72881 |
| Ras signaling pathway |  | 5.97829 |  |  |
| Regulation of lipolysis in adipocytes |  |  |  |  |
| Relaxin signaling pathway |  | 3.45565 |  | 2.66776 |
| Renal cell carcinoma |  |  |  | 3.15862 |
| Renin secretion |  | 1.74932 |  |  |
| Renin-angiotensin system |  |  |  |  |
| Retinol metabolism |  |  |  |  |
| Retrograde endocannabinoid signaling |  | 1.56051 | 1.4852 |  |
| Rheumatoid arthritis |  | 3.22458 |  |  |
| RIG-I-like receptor signaling pathway |  | 1.88787 |  |  |
| Salivary secretion |  | 4.5534 |  |  |
| Salmonella infection |  | 3.0341 |  |  |
| Serotonergic synapse | 1.76345 | 3.84495 |  |  |
| Shigellosis |  | 2.14089 |  | 2.27104 |
| Sphingolipid signaling pathway |  | 3.68899 |  |  |
| Steroid hormone biosynthesis |  |  |  |  |
| Synaptic vesicle cycle |  |  |  |  |
| Systemic lupus erythematosus | 6.65037 |  |  |  |
| T cell receptor signaling pathway |  | 6.12446 |  | 2.73081 |
| Th1 and Th2 cell differentiation |  | 7.43671 |  |  |
| Th17 cell differentiation |  | 12.8393 |  |  |
| Thyroid cancer |  | 3.77108 |  |  |
| Thyroid hormone signaling pathway |  | 4.18308 |  | 7.94734 |
| Thyroid hormone synthesis |  | 1.37939 |  |  |
| Tight junction |  | 2.22674 |  |  |
| TNF signaling pathway |  | 7.43671 |  | 1.61896 |
| Toll-like receptor signaling pathway |  | 3.25804 |  |  |
| Toxoplasmosis |  | 6.73391 |  |  |
| Transcriptional misregulation in cancer | 1.80498 | 1.53866 |  | 2.74949 |
| Tuberculosis |  | 7.54109 |  |  |
| Type I diabetes mellitus |  | 5.66431 |  |  |
| Type II diabetes mellitus |  | 1.53204 |  | 1.34821 |
| Vascular smooth muscle contraction |  | 3.89256 |  |  |
| VEGF signaling pathway |  | 2.95273 |  | 2.75989 |
| Vibrio cholerae infection |  |  |  |  |
| Viral carcinogenesis | 2.22839 | 3.49451 |  | 7.61078 |
| Viral myocarditis |  | 6.87677 |  |  |
| Wnt signaling pathway |  |  |  | 1.89518 |
| Adherens junction |  | 3.01133 |  | 5.23963 |
| Antifolate resistance |  |  |  |  |
| Axon guidance |  |  |  | 3.90951 |
| Basal transcription factors |  |  |  |  |
| Base excision repair |  |  |  |  |
| Bladder cancer |  |  |  | 2.17635 |
| Breast cancer |  |  |  | 8.03715 |
| Cell cycle |  |  |  | 11.1254 |
| DNA replication |  |  |  |  |
| Fanconi anemia pathway |  |  |  |  |
| Focal adhesion |  | 3.27787 |  | 4.44275 |
| Gastric cancer |  | 2.06766 |  | 3.14097 |
| Hippo signaling pathway |  |  |  | 4.19189 |
| Homologous recombination |  |  |  | 1.32375 |
| Lysine degradation |  |  |  |  |
| MicroRNAs in cancer |  |  |  | 2.84589 |
| Mismatch repair |  |  |  | 1.61043 |
| mTOR signaling pathway | 3.18412 |  |  | 2.4644 |
| Notch signaling pathway |  |  |  | 5.69534 |
| Nucleotide excision repair |  |  |  |  |
| One carbon pool by folate |  |  |  |  |
| Pathogenic Escherichia coli infection |  |  |  | 5.83004 |
| Progesterone-mediated oocyte maturation | 1.98663 |  |  | 4.44275 |
| Proteasome | 2.59014 |  | 8.80164 |  |
| Regulation of actin cytoskeleton |  | 2.27223 |  | 1.7104 |
| Ribosome |  |  | 11.5329 | 13 |
| RNA polymerase |  |  |  |  |
| RNA transport |  |  |  | 10.2396 |
| Signaling pathways regulating pluripotency of stem cells |  |  |  | 4.12397 |
| Small cell lung cancer |  | 2.27504 |  | 4.9607 |
| Spliceosome |  |  |  | 10.0594 |
| Ubiquitin mediated proteolysis | 2.65642 |  |  | 10.1117 |
| JAK-STAT signaling pathway |  |  |  |  |
| TGF-beta signaling pathway |  |  |  | 2.09746 |
| Cortisol synthesis and secretion |  | 1.44368 |  |  |
| mRNA surveillance pathway |  |  |  | 4.69038 |
| Oxidative phosphorylation |  |  | 4.25944 |  |
| Thermogenesis |  |  | 2.02445 |  |
| Alanine, aspartate and glutamate metabolism |  | 2.81488 |  |  |
| Arginine and proline metabolism |  | 2.62505 |  |  |
| Asthma |  | 2.60385 |  |  |
| Autoimmune thyroid disease |  | 3.39507 |  |  |
| Biosynthesis of amino acids |  | 2.84113 |  |  |
| Carbon metabolism |  | 2.33251 |  |  |
| Cell adhesion molecules (CAMs) |  | 3.21036 |  |  |
| Fructose and mannose metabolism |  | 1.41954 |  |  |
| Glycine, serine and threonine metabolism |  | 1.44695 |  |  |
| Glycolysis / Gluconeogenesis |  | 1.98295 |  |  |
| Hematopoietic cell lineage |  | 3.24156 |  |  |
| Intestinal immune network for IgA production |  | 2.22345 |  |  |
| Malaria |  | 2.22345 |  |  |
| PPAR signaling pathway |  | 1.70875 |  |  |
| Staphylococcus aureus infection |  | 2.22345 |  |  |
| Tyrosine metabolism |  | 1.68109 |  |  |
| Valine, leucine and isoleucine degradation |  | 1.83621 |  |  |
| Vitamin B6 metabolism |  | 1.63992 |  |  |
| Cytosolic DNA-sensing pathway |  |  |  |  |
| ECM-receptor interaction |  |  |  |  |
| SNARE interactions in vesicular transport |  |  |  |  |

**Supplementary Table S3: Regional specificities of DGN1-DGN4 in BrainSpan Atlas.** The -log_10_p-values of enrichment of the DGNs in BrainSpan regions. Note that this is the data underlying Fig. 3a.

| Brain region | DGN1 | DGN2 | DGN3 | DGN4 |
| --- | --- | --- | --- | --- |
| Amygdaloid complex | 2.25564 | 7 | 2.35141 | 12.4739 |
| Caudal ganglionic eminence | 2.4881 | 0 | 2.29863 | 15 |
| Cerebellar cortex | 0 | 7 | 1.86957 | 8.62588 |
| Cerebellum | 5.68963 | 6.2 | 2.35141 | 15 |
| Dorsal thalamus | 6.61631 | 3.234 | 2.35141 | 15 |
| Dorsolateral prefrontal cortex | 5.77068 | 7 | 2.35141 | 5.44105 |
| Hippocampus (hippocampal formation) | 2.01805 | 7 | 2.29863 | 14.882 |
| Lateral ganglionic eminence | 3.85658 | 0 | 2.35141 | 15 |
| Medial ganglionic eminence | 2.83233 | 1.56 | 2.35141 | 15 |
| Mediodorsal nucleus of thalamus | 2.05219 | 7 | 2.35141 | 3.27968 |
| Occipital neocortex | 3.68651 | 0 | 2.35141 | 15 |
| Orbital frontal cortex | 6.19138 | 7 | 2.35029 | 5.46691 |
| Parietal neocortex | 5.18493 | 0 | 2.35141 | 15 |
| Posterior (caudal) superior temporal cortex (area 22c) | 5.752 | 7 | 2.35141 | 2.91725 |
| Posteroventral (inferior) parietal cortex | 7.10336 | 7 | 2.35141 | 3.41094 |
| Primary auditory cortex (core) | 6.12151 | 7 | 2.35141 | 2.68275 |
| Primary motor cortex (area m1, area 4) | 6.61802 | 7 | 2.35141 | 3.54613 |
| Primary motor-sensory cortex (samples) | 7.10336 | 2.886 | 2.35141 | 15 |
| Primary somatosensory cortex (area s1, areas 3,1,2) | 7.10336 | 7 | 2.35141 | 3.18773 |
| Primary visual cortex (striate cortex, area v1/17) | 5.54477 | 7 | 2.35141 | 4.74951 |
| Striatum | 3.79596 | 7 | 2.35141 | 4.89582 |
| Temporal neocortex | 7.10336 | 0 | 2.35141 | 15 |
| Upper (rostral) rhombic lip | 5.68963 | 5.346 | 1.92107 | 15 |
| Ventrolateral prefrontal cortex | 6.41912 | 7 | 2.35141 | 3.93704 |

**Supplementary Table S4: Regional specificities of DGN1-DGN4 in GTEx.** The -log_10_p-values of enrichment of the DGNs in GTEx regions. Note that this is the data underlying Fig. 3b.

| Brain region | DGN1 | DGN2 | DGN3 | DGN4 |
| --- | --- | --- | --- | --- |
| Amygdala | 0.9064 | 14.8406 | 2.23074 | 0 |
| Anterior cingulate cortex (BA24) | 2.65049 | 12.0229 | 2.29773 | 0 |
| Caudate (basal ganglia) | 1.15483 | 13.2451 | 2.23074 | 0 |
| Cerebellar Hemisphere | 1.08827 | 0.00104 | 2.06989 | 4.24807 |
| Cerebellum | 0.68288 | 0.00104 | 1.54204 | 4.18299 |
| Cortex | 3.70432 | 11.1627 | 1.84091 | 0 |
| Frontal Cortex (BA9) | 7.46726 | 8.3934 | 2.29773 | 0 |
| Hippocampus | 1.52203 | 13.6102 | 2.39463 | 0 |
| Hypothalamus | 1.2148 | 10.9598 | 2.05854 | 0 |
| Nucleus accumbens (basal ganglia) | 1.99762 | 10.5989 | 2.23074 | 0 |
| Putamen (basal ganglia) | 0.87621 | 14.3178 | 2.29773 | 0 |
| Spinal cord (cervical c-1) | 0.46505 | 11.6527 | 2.0614 | 0 |
| Substantia nigra | 0.78757 | 15 | 2.39463 | 0 |

**Supplementary Table S5: Regional specificities of DGN1-DGN4 in prenatal microarray dataset.** The -log_10_p-values of enrichment of the DGNs in prenatal structures. Note that this is the data underlying Supplementary Fig. S3.

| Prenatal structure | DGN1 | DGN2 | DGN3 | DGN4 |
| --- | --- | --- | --- | --- |
| Agranular insular cortex (area iag) | 10.35264 |  |  |  |
| Caudal division of ipc (area 39) | 4.910236 |  | 14 |  |
| Caudal presubiculum (postsubiculum) | 4.323553 |  |  |  |
| Caudal prosubiculum | 12.46378 |  |  |  |
| Central nuclear group | 11.39995 |  |  |  |
| Cp in caudal hippocampus | 14 |  | 4.929556 |  |
| Cp in caudal subicular cortex | 10.06896 |  | 3.485798 |  |
| Deep layers of caudal entorhinal cortex | 4.217843 |  |  |  |
| Dorsal claustrum | 2.853779 |  |  |  |
| Dorsal motor nucleus of the vagus (vagal nucleus) | 6.075933 | 13 |  |  |
| Dorsal subdivision of vlc | 4.260705 |  |  |  |
| Facial nucleus | 13.54297 | 13 | 11.41627 |  |
| Indusium griseum | 4.108596 |  | 7.244026 | 15 |
| Inferior colliculus | 8.469032 |  |  |  |
| Inner cp in dorsolateral prefrontal cortex | 3.37732 |  |  |  |
| Inner cp in medial temporal-occipital cortex | 6.10968 |  |  |  |
| Inner cp in orbital frontal cortex | 4.768021 |  | 6.573863 |  |
| Inner cp in posteror frontal cortex (motor cortex) | 2.475513 |  |  |  |
| Lateral nucleus of pulvinar | 3.497996 | 2.665144 | 10.74072 |  |
| Lateral orbital frontal cortex (area 12/47) | 4.498886 |  | 14 |  |
| Lateral subdivision of area 9 | 7.781123 |  | 11.56265 | 15 |
| Layer iiia of caudal entorhinal cortex | 6.230497 |  |  |  |
| Layer iiib of caudal entorhinal cortex | 5.557458 |  |  |  |
| Layer v of caudal entorhinal cortex | 14 |  |  |  |
| Layer vi of caudal entorhinal cortex | 3.963172 |  |  |  |
| Magnocellular division of va | 5.764194 |  | 5.658467 |  |
| Medioventral part of putamen | 7.357951 |  |  | 12.64786 |
| Multiform (lateral) division of md | 4.790887 |  |  |  |
| Mz in temporal polar cortex | 9.558965 |  |  |  |
| Outer cp in (rostral) midinferior temporal cortex (area 36) | 12.79477 |  |  |  |
| Outer cp in frontal polar cortex | 4.760701 |  | 3.890556 | 15 |
| Outer cp in medial temporal-occipital cortex | 2.595525 |  | 14 | 15 |
| Outer cp in subgenual (subcallosal) cingulate cortex | 10.35264 |  |  |  |
| Outer cp in ventrolateral prefrontal cortex | 6.107733 |  |  |  |
| Polymorphic layer of caudal subiculum | 4.749434 |  |  |  |
| Polymorphic layer of rostral subiculum | 6.727277 |  | 5.333332 |  |
| Principal sensory nucleus of trigeminal nerve | 6.033539 | 3.385335 |  |  |
| Pyramidal layer of caudal subiculum | 14 |  |  |  |
| Sp in inferolateral temporal cortex | 2.031704 |  |  |  |
| Sp in medial temporal-occipital cortex | 5.981216 |  |  |  |
| Sp in midlateral temporal cortex | 3.468981 |  |  |  |
| Sp in rostral midinferior temporal cortex (area 36) | 8.39869 |  |  |  |
| Sp in ventrolateral prefrontal cortex | 1.450114 |  |  |  |
| Stratum pyramidale of caudal ca1 | 11.39995 |  |  |  |
| Stratum pyramidale of caudal ca3 | 9.473751 |  |  |  |
| Subthalamic nucleus | 8.376286 |  |  |  |
| Upper limb region of m1 | 6.075933 |  | 13.175 | 15 |
| Ventral pallidus | 4.497791 |  |  |  |
| Abducens nucleus |  | 13 |  |  |
| Area postrema |  | 13 |  |  |
| Central nucleus of inferior colliculus |  | 13 | 12.52981 |  |
| Choroid plexus of the fourth ventricle |  | 13 |  |  |
| Corpus callosum |  | 13 |  |  |
| Gigantocellular reticular nuclei |  | 13 |  |  |
| Hypoglossal nucleus |  | 13 |  |  |
| Inner sz in midlateral extrastriate cortex |  | 12.87128 |  |  |
| Intercalated nucleus of medulla |  | 13 |  |  |
| Internal segment of globus pallidus |  | 12.01816 |  |  |
| Lateral reticular nucleus (principal part) |  | 11.5107 |  |  |
| Layer I of piriform cortex |  | 13 |  |  |
| Medial parabrachial nucleus |  | 5.01166 |  |  |
| Medial subdivision of central nucleus |  | 9.23905 |  |  |
| Motor nucleus of trigeminal nerve |  | 13 |  |  |
| Nucleus coeruleus |  | 11.47903 |  |  |
| Oculomotor nucleus |  | 13 |  |  |
| Parabigeminal nucleus |  | 12.02344 |  |  |
| Parafascicular nucleus of thalamus |  | 4.913783 |  |  |
| Periventricular nucleus, preoptic portion |  | 2.361611 |  |  |
| Pineal body |  | 13 |  |  |
| Raphe magnus nucleus |  | 13 |  |  |
| Raphe obscurus nucleus |  | 13 |  |  |
| Retroparafascicular area of thalamus |  | 11.09153 |  |  |
| Rostral subdivision of medial nucleus |  | 3.096086 |  |  |
| Rostral ventral respiratory cell group |  | 9.218532 |  |  |
| Sg in caudal perirhinal cortex |  | 13 |  |  |
| Sg in dorsomedial parietal cortex (area 7m) |  | 12.57169 |  |  |
| Sg in dysgranular insular cortex |  | 11.38622 |  |  |
| Sg in granular insular cortex |  | 8.656296 |  |  |
| Sg in inferolateral temporal cortex |  | 8.6674 |  |  |
| Sg in midlateral temporal cortex |  | 13 |  |  |
| Sg in primary somatosensory cortex |  | 12.85874 |  |  |
| Sg in rostral entorhinal cortex |  | 8.230926 |  |  |
| Sg in rostral perirhinal cortex |  | 13 |  |  |
| Spinal (inferior) vestibular nucleus |  | 8.870439 |  |  |
| Substantia nigra, reticular part |  | 8.228398 |  |  |
| Supraoptic nucleus |  | 13 |  |  |
| Ventricular (matrix) zone of pons |  | 13 |  |  |
| Vz in midcingulate neocortex |  | 13 |  |  |
| Vz in midlateral extrastriate cortex (area 19) |  | 8.303652 |  | 15 |
| Vz in thalamic region |  | 9.468291 |  |  |
| Vz in ventromedial extrastriate cortex (vp) |  | 2.488986 |  | 15 |
| Anterior nucleus of pulvinar |  |  | 1.675409 |  |
| Basal nucleus of meynert |  |  | 5.432209 |  |
| Central portion of gpi |  |  | 2.96457 |  |
| Dorsal lateral geniculate nucleus |  |  | 14 |  |
| Granular layer of caudal dentate gyrus |  |  | 10.01681 |  |
| Head and face region of m1 |  |  | 11.41977 | 15 |
| Inner cp in dorsomedial frontal cortex |  |  | 14 |  |
| Laterodorsal subdivision of area 8 |  |  | 14 |  |
| Layer ii of area 35c |  |  | 14 | 13.83556 |
| Layer iii of area 35c |  |  | 5.879821 |  |
| Layer iiiu of area 35c |  |  | 6.425888 |  |
| Layer vi of area 35c |  |  | 6.657479 |  |
| Lower limb region of m1 |  |  | 14 |  |
| Mge-lateral region |  |  | 4.163094 | 15 |
| Olfactory bulb |  |  | 4.255347 |  |
| Outer cp in caudal cingulate cortex |  |  | 6.193569 |  |
| Outer cp in dorsolateral prefrontal cortex |  |  | 6.573863 |  |
| Outer cp in dorsomedial frontal cortex |  |  | 13.58436 |  |
| Outer cp in dorsomedial parietal cortex (area 7m) |  |  | 8.927383 |  |
| Outer cp in midlateral extrastriate cortex |  |  | 7.450862 | 15 |
| Outer cp in posteror frontal cortex (motor cortex) |  |  | 5.027196 |  |
| Outer sz in dorsomedial frontal cortex |  |  | 6.531594 |  |
| Outer sz in orbital frontal cortex |  |  | 4.417391 | 15 |
| Polysensory temporal cortex (area 22p) |  |  | 9.847345 |  |
| Rostral (anterior) extramural migratory stream |  |  | 13.94089 |  |
| Rostral division of ofci (area 11) |  |  | 14 |  |
| Stratum radiatum of rostral ca1 |  |  | 8.710924 |  |
| Substantia nigra, compact part |  |  | 6.573863 |  |
| Superficial layers of caudal presubiculum |  |  | 11.11732 | 11.70783 |
| Transient purkinje cell clusters |  |  | 5.321345 |  |
| Caudal ganglionic eminence |  |  |  | 15 |
| Inner cp in posterosuperior (dorsal) parietal cortex |  |  |  | 1.821685 |
| Inner portion of lateral ganglionic eminence |  |  |  | 15 |
| Inner portion of medial ganglionic eminence |  |  |  | 15 |
| Inner sz in caudal midinferior temporal cortex (area tf) |  |  |  | 15 |
| Inner sz in dorsolateral prefrontal cortex |  |  |  | 8.885322 |
| Inner sz in dorsomedial extrastriate cortex |  |  |  | 15 |
| Inner sz in primary somatosensory cortex |  |  |  | 6.524416 |
| Inner sz in primary visual cortex |  |  |  | 15 |
| Inner sz in superolateral temporal cortex |  |  |  | 15 |
| Lateral ganglionic eminence |  |  |  | 15 |
| Lge-vz border region |  |  |  | 15 |
| Medial ganglionic eminence |  |  |  | 15 |
| Mz in inferolateral temporal cortex |  |  |  | 1.847834 |
| Mz in midlateral extrastriate cortex (area 19) |  |  |  | 1.648784 |
| Outer cp in posteroinferior (ventral) parietal cortex |  |  |  | 11.98923 |
| Outer cp in primary auditory cortex |  |  |  | 15 |
| Outer cp in superolateral temporal cortex |  |  |  | 15 |
| Outer cp in ventromedial extrastriate cortex |  |  |  | 15 |
| Outer sz in dorsomedial extrastriate cortex |  |  |  | 15 |
| Outer sz in dorsomedial parietal cortex (area 7m) |  |  |  | 15 |
| Outer sz in midlateral temporal cortex |  |  |  | 14.62215 |
| Outer sz in posteroinferior (ventral) parietal cortex |  |  |  | 15 |
| Outer sz in posteror frontal cortex (motor cortex) |  |  |  | 15 |
| Outer sz in superolateral temporal cortex |  |  |  | 15 |
| Outer sz in ventrolateral prefrontal cortex |  |  |  | 15 |
| Rostral migratory stream |  |  |  | 15 |
| Sg in posterior frontal cortex (motor cortex) |  |  |  | 9.449943 |
| Sg in ventrolateral prefrontal cortex |  |  |  | 6.243691 |
| Sz in caudal cingulate cortex |  |  |  | 15 |
| Vz in caudal cingulate neocortex |  |  |  | 15 |
| Vz in caudal subicular cortex |  |  |  | 15 |
| Vz in dorsomedial extrastriate cortex (v2) |  |  |  | 15 |
| Vz in frontal polar cortex |  |  |  | 15 |
| Vz in inferolateral temporal cortex |  |  |  | 15 |
| Vz in primary visual cortex |  |  |  | 15 |
| Vz in superolateral temporal cortex |  |  |  | 15 |
| Vz in ventrolateral prefrontal cortex |  |  |  | 15 |
| Vz in caudal midinferior temporal cortex (area tf) |  |  |  |  |
| Vz in dorsomedial parietal cortex (area 7m) |  |  |  |  |
| Outer sz in caudal midinferior temporal cortex (area tf) |  |  |  |  |
| Vz in dorsolateral prefrontal cortex |  |  |  |  |
| Vz in lateral temporal-occipital cortex |  |  |  |  |
| Vz in posteror frontal cortex (motor cortex) |  |  |  |  |
| Inner sz in dorsomedial parietal cortex (area 7m) |  |  |  |  |
| Inner sz in medial temporal-occipital cortex |  |  |  |  |
| Outer sz in posterosuperior (dorsal) parietal cortex |  |  |  |  |
| Outer sz in midlateral extrastriate cortex |  |  |  |  |
| Inner cp in ventrolateral prefrontal cortex |  |  |  |  |
| Vz in orbital frontal cortex |  |  |  |  |
| Vz in posteroinferior (ventral) parietal cortex |  |  |  |  |
| Outer sz in inferolateral temporal cortex |  |  |  |  |
| Intermediate portion of dfc (area 9/46) |  |  |  |  |
| Sg in lateral temporal-occipital cortex |  |  |  |  |
| Outer portion of lateral ganglionic eminence |  |  |  |  |
| Outer portion of medial ganglionic eminence |  |  |  |  |
| Vz in midlateral temporal cortex |  |  |  |  |
| Outer cp in rostral cingulate cortex |  |  |  |  |
| Pontobulbar body |  |  |  |  |
| Outer sz in primary somatosensory cortex |  |  |  |  |
| Vz in medial temporal-occipital cortex |  |  |  |  |
| Outer sz in primary visual cortex |  |  |  |  |
| Inner sz in dorsomedial frontal cortex |  |  |  |  |
| Sz in subgenual cingulate cortex |  |  |  |  |
| Inner sz in frontal polar cortex |  |  |  |  |
| Outer sz in lateral temporal-occipital cortex |  |  |  |  |
| Iz in posterosuperior (dorsal) parietal cortex |  |  |  |  |
| Vz in posterior parahippocampal cortex |  |  |  |  |
| Outer sz in dorsolateral prefrontal cortex |  |  |  |  |
| Inner sz in posterior parahippocampal cortex |  |  |  |  |
| Vz in subgenual cingulate neocortex |  |  |  |  |
| Inner sz in posterosuperior (dorsal) parietal cortex |  |  |  |  |
| Inner sz in ventromedial extrastriate cortex |  |  |  |  |
| Outer sz in frontal polar cortex |  |  |  |  |
| Vz in posterosuperior (dorsal) parietal cortex |  |  |  |  |
| Sz in midcingulate cortex |  |  |  |  |
| Outer sz in ventromedial extrastriate cortex |  |  |  |  |
| Outer cp in lateral temporal-occipital cortex |  |  |  |  |
| Medial subdivision of area 10 |  |  |  |  |
| Inner sz in orbital frontal cortex |  |  |  |  |
| Outer cp in dorsomedial extrastriate cortex |  |  |  |  |
| Outer sz in medial temporal-occipital cortex |  |  |  |  |
| Inner cp in caudal cingulate cortex |  |  |  |  |
| Vz in caudal hippocampal proper |  |  |  |  |
| Inner sz in ventrolateral prefrontal cortex |  |  |  |  |
| Caudal (posterior) extramural migratory stream |  |  |  |  |
| Outer cp in caudal midinferior temporal cortex (area tf) |  |  |  |  |
| Inner sz in posteroinferior (ventral) parietal cortex |  |  |  |  |
| Sz in posterior parahippocampal cortex |  |  |  |  |
| Mz in lateral temporal-occipital cortex |  |  |  |  |
| Vz in primary somatosensory cortex |  |  |  |  |
| Inner cp in posteroinferior (ventral) parietal cortex |  |  |  |  |
| Iz in dorsomedial frontal cortex |  |  |  |  |
| Sg in frontal polar cortex |  |  |  |  |
| Mz in posterodorsal (superior) parietal cortex |  |  |  |  |
| Inner cp in primary auditory cortex |  |  |  |  |
| Outer cp in temporal polar cortex |  |  |  |  |
| Mz in frontal polar cortex |  |  |  |  |
| Inner cp in primary somatosensory cortex |  |  |  |  |
| Inner cp in frontal polar cortex |  |  |  |  |
| Outer sz in posterior parahippocampal cortex |  |  |  |  |
| Mz in caudal cingulate cortex |  |  |  |  |
| Premotor cortex (area 6) |  |  |  |  |
| Mz in medial temporal-occipital cortex |  |  |  |  |
| Outer cp in inferolateral temporal cortex |  |  |  |  |
| Sp in frontal polar cortex |  |  |  |  |
| Sg in midlateral extrastriate cortex (area 19) |  |  |  |  |
| Inner cp in superolateral temporal cortex |  |  |  |  |
| Iz in dorsomedial parietal cortex (area 7m) |  |  |  |  |
| Sg in temporal polar cortex |  |  |  |  |
| Vz in subcallosal region |  |  |  |  |
| Outer cp in posterosuperior (dorsal) parietal cortex |  |  |  |  |
| Mz in ventrolateral prefrontal cortex |  |  |  |  |
| Inner cp in midlateral extrastriate cortex |  |  |  |  |
| Mz in orbital frontal cortex |  |  |  |  |
| Mz in dorsomedial frontal cortex |  |  |  |  |
| Inner sz in midlateral temporal cortex |  |  |  |  |
| External granular (germinal) layer of upper rhombic lip |  |  |  |  |
| Upper (rostral) rhombic lip |  |  |  |  |
| Iz in rostral cingulate cortex |  |  |  |  |
| Vz in rostral cingulate neocortex |  |  |  |  |
| Inner cp in posterior parahippocampal cortex |  |  |  |  |
| Sz in medial temporal-occipital cortex |  |  |  |  |
| Pedunculo(pontine) tegmental nucleus |  |  |  |  |
| Cp in temporal polar cortex |  |  |  |  |
| Cochlear nuclei |  |  |  |  |
| Solitary nucleus |  |  |  |  |
| Medial superior olive |  |  |  |  |
| Red nucleus |  |  |  |  |
| Paraventricular nucleus |  |  |  |  |
| Reticulotegmental nucleus |  |  |  |  |
| Interpeduncular nucleus |  |  |  |  |
| Centromedian nucleus of thalamus |  |  |  |  |
| Lateral dorsal nucleus of thalamus |  |  |  |  |
| Cuneate nucleus |  |  |  |  |
| Medial septal nucleus |  |  |  |  |
| Stratum pyramidale of rostral ca2 |  |  |  |  |
| Olfactory part of entorhinal cortex |  |  |  |  |
| Ventral posterior lateral nucleus |  |  |  |  |
| Medial nucleus of pulvinar |  |  |  |  |
| Medial geniculate nuclei |  |  |  |  |
| Stratum pyramidale of rostral ca1 |  |  |  |  |
| Stratum pyramidale of caudal ca2 |  |  |  |  |
| Gracile nucleus |  |  |  |  |
| Inferior nucleus of pulvinar |  |  |  |  |
| Caudal putamen |  |  |  |  |
| Anterior amygdaloid area |  |  |  |  |
| Edinger-westphal nucleus (accessory oculomotor nucleus) |  |  |  |  |
| Parataenial nucleus of thalamus |  |  |  |  |
| Periaqueductal gray substance, dorsolateral portion |  |  |  |  |
| Basal ventral medial nucleus |  |  |  |  |
| Rostral division of vl |  |  |  |  |
| Rostral putamen |  |  |  |  |
| Subparafascicular nucleus of thalamus |  |  |  |  |
| Nucleus of diagonal band |  |  |  |  |
| Ventral medial nucleus of thalamus |  |  |  |  |
| Lateral hypothalamic area, anterior part |  |  |  |  |
| Inferior olive, principal nucleus |  |  |  |  |
| Superior colliculus |  |  |  |  |
| Limitans nucleus |  |  |  |  |
| Anterodorsal nucleus of thalamus |  |  |  |  |
| Callosal sling |  |  |  |  |
| Layer v of area 35c |  |  |  |  |
| Ventral lateral nucleus of thalamus |  |  |  |  |
| Midbrain reticular formation |  |  |  |  |
| Medial vestibular nucleus |  |  |  |  |
| Cerebellar vermis |  |  |  |  |
| External segment of globus pallidus |  |  |  |  |
| Endopiriform nucleus |  |  |  |  |
| Lateral subdivision of bnst |  |  |  |  |
| Parasolitary nucleus |  |  |  |  |
| Interpositus (intermediate) nucleus |  |  |  |  |
| Zona incerta |  |  |  |  |
| Central part of cel |  |  |  |  |
| Prepositus hypoglossal nucleus |  |  |  |  |
| Posterior hypothalamic nucleus |  |  |  |  |
| Amygdalostriatal transition area |  |  |  |  |
| Lateral hypothalamic area, tuberal part |  |  |  |  |
| Anterior cortical nucleus |  |  |  |  |
| Lateral parabrachial nucleus |  |  |  |  |
| Core of nucleus accumbens |  |  |  |  |
| Dorsomedial hypothalamic nucleus |  |  |  |  |
| Reticular nucleus of thalamus |  |  |  |  |
| Ventral claustrum |  |  |  |  |
| Spinal trigeminal nucleus |  |  |  |  |
| Midline nuclear complex |  |  |  |  |
| Layer ii of piriform cortex |  |  |  |  |
| Sg in midinferior temporal cortex |  |  |  |  |
| Pretectal nuclear complex |  |  |  |  |
| Sp in dysgranular insular cortex |  |  |  |  |
| Layer iii of piriform cortex |  |  |  |  |
| Sg in caudal entorhinal cortex |  |  |  |  |
| Dorsal cochlear nucleus |  |  |  |  |
| Pyramidal layer of rostral subiculum |  |  |  |  |
| Inferior olive, dorsal nucleus |  |  |  |  |
| Olfactory tubercle |  |  |  |  |
| Ventromedial hypothalamic nucleus |  |  |  |  |

**Supplementary Table S6: Regional specificities of DGN1-DGN4, and LBD and 22q11del interactomes, in BrainSpan Atlas.** The -log_10_p-values of enrichment of the DGNs in BrainSpan regions. Note that this is the data underlying Fig. 4a.

| Brain region | DGN1 | DGN2 | DGN3 | DGN4 | LBD | 22q11del |
| --- | --- | --- | --- | --- | --- | --- |
| amygdaloid complex | 2.25564 | 7 | 2.35141 | 12.4739 | 0.51135 | 2.75105 |
| anterior (rostral) cingulate (medial prefrontal) cortex |  |  |  |  | 0.06199 | 0 |
| caudal ganglionic eminence | 2.4881 | 0 | 2.29863 | 15 | 0.12831 | 8.45209 |
| cerebellar cortex | 0 | 7 | 1.86957 | 8.62588 | 0.51135 | 2.128 |
| cerebellum | 5.68963 | 6.20014 | 2.35141 | 15 | 0.51135 | 4.25263 |
| Dorsal thalamus | 6.61631 | 3.23428 | 2.35141 | 15 | 0.51135 | 2.34887 |
| dorsolateral prefrontal cortex | 5.77068 | 7 | 2.35141 | 5.44105 | 1.00427 | 2.10218 |
| hippocampus (hippocampal formation) | 2.01805 | 7 | 2.29863 | 14.882 | 1.00427 | 5.32143 |
| lateral ganglionic eminence | 3.85658 | 0 | 2.35141 | 15 |  | 0 |
| medial ganglionic eminence | 2.83233 | 1.55958 | 2.35141 | 15 | 0.51135 | 8.36843 |
| mediodorsal nucleus of thalamus | 2.05219 | 7 | 2.35141 | 3.27968 | 0.51135 | 8.19673 |
| occipital neocortex | 3.68651 | 0 | 2.35141 | 15 | 0.99413 | 2.19577 |
| orbital frontal cortex | 6.19138 | 7 | 2.35029 | 5.46691 | 1.00427 | 8.48381 |
| parietal neocortex | 5.18493 | 0 | 2.35141 | 15 | 1.00427 | 2.15411 |
| posterior (caudal) superior temporal cortex (area 22c) | 5.752 | 7 | 2.35141 | 2.91725 | 1.00427 | 8.19673 |
| posteroventral (inferior) parietal cortex | 7.10336 | 7 | 2.35141 | 3.41094 | 1.00427 | 1.77247 |
| primary auditory cortex (core) | 6.12151 | 7 | 2.35141 | 2.68275 | 1.00427 | 1.32444 |
| primary motor cortex (area M1, area 4) | 6.61802 | 7 | 2.35141 | 3.54613 | 1.00427 | 1.77583 |
| primary motor-sensory cortex (samples) | 7.10336 | 2.88572 | 2.35141 | 15 | 1.00427 | 1.97062 |
| primary somatosensory cortex (area S1, areas 3,1,2) | 7.10336 | 7 | 2.35141 | 3.18773 | 1.00427 | 6.19874 |
| primary visual cortex (striate cortex, area V1/17) | 5.54477 | 7 | 2.35141 | 4.74951 | 1.00427 | 1.61964 |
| striatum | 3.79596 | 7 | 2.35141 | 4.89582 | 0.51135 | 1.78323 |
| temporal neocortex | 7.10336 | 0 | 2.35141 | 15 | 1.00427 | 1.75318 |
| upper (rostral) rhombic lip | 5.68963 | 5.34634 | 1.92107 | 15 | 1.00427 | 6.74184 |
| ventrolateral prefrontal cortex | 6.41912 | 7 | 2.35141 | 3.93704 | 1.00427 | 4.04875 |
| inferolateral temporal cortex (area TEv, area 20) |  |  |  |  | 0.51135 | 1.78323 |

**Supplementary Table S7: Regional specificities of DGN1-DGN4, and LBD and 22q11del interactomes, in GTEx.** The -log_10_p-values of enrichment of the DGNs in GTEx regions. Note that this is the data underlying Fig. 4b.

| Brain region | DGN1 | DGN2 | DGN3 | DGN4 | LBD | 22q11del |
| --- | --- | --- | --- | --- | --- | --- |
| Amygdala | 0.9064 | 14.8406 | 2.23074 | 0 | 1.15861 | 0.60199 |
| Anterior cingulate cortex (BA24) | 2.65049 | 12.0229 | 2.29773 | 0 | 1.43993 | 0.42323 |
| Caudate (basal ganglia) | 1.15483 | 13.2451 | 2.23074 | 0 | 1.11934 | 0.59184 |
| Cerebellar Hemisphere | 1.08827 | 0.00104 | 2.06989 | 4.24807 | 0.68162 | 0.60199 |
| Cerebellum | 0.68288 | 0.00104 | 1.54204 | 4.18299 | 0.68162 | 0.59184 |
| Cortex | 3.70432 | 11.1627 | 1.84091 | 0 | 1.15861 | 0.36455 |
| Frontal Cortex (BA9) | 7.46726 | 8.3934 | 2.29773 | 0 | 1.43993 | 0.36455 |
| Hippocampus | 1.52203 | 13.6102 | 2.39463 | 0 | 1.11934 | 0.60199 |
| Hypothalamus | 1.2148 | 10.9598 | 2.05854 | 0 | 1.09903 | 0.68744 |
| Nucleus accumbens (basal ganglia) | 1.99762 | 10.5989 | 2.23074 | 0 | 1.15861 | 0.59184 |
| Putamen (basal ganglia) | 0.87621 | 14.3178 | 2.29773 | 0 | 1.15861 | 0.43072 |
| Spinal cord (cervical c-1) | 0.46505 | 11.6527 | 2.0614 | 0 | 0.57104 | 1.91396 |
| Substantia nigra | 0.78757 | 15 | 2.39463 | 0 | 0.79547 | 0.78133 |

**Supplementary Table S8: Synaptic signaling specificities of DGN1-DGN4 in KEGG.** The -log_10_p-values of enrichment of the DGNs in five synaptic signaling pathways. Note that this is the data underlying Fig. 3c.

| Synaptic signaling pathway | DGN1 | DGN2 | DGN3 | DGN4 |
| --- | --- | --- | --- | --- |
| Cholinergic synapse | 0.00083 | 0.17522 | 0.03279 | 0.03443 |
| Dopaminergic synapse | 0.52943 | 0.21999 | 0.70567 | 0.03443 |
| GABAergic synapse | 0.00083 | 0.21999 | 0.03279 | 0.03443 |
| Glutamatergic synapse | 0.00083 | 0.23982 | 0.03279 | 0.03443 |
| Serotonergic synapse | 0.00083 | 0.21999 | 0.03279 | 0.03443 |

**Supplementary References**

1 Piñero, J. *et al.* DisGeNET: a comprehensive platform integrating information on human disease-associated genes and variants. *Nucleic acids research*, gkw943 (2016).

2 Trubetskoy, V. *et al.* Mapping genomic loci implicates genes and synaptic biology in schizophrenia. *Nature* **604**, 502-508 (2022).

3 Singh, T. *et al.* Rare coding variants in ten genes confer substantial risk for schizophrenia. *Nature* **604**, 509-516 (2022).

4 Nalls, M. A. *et al.* Identification of novel risk loci, causal insights, and heritable risk for Parkinson's disease: a meta-analysis of genome-wide association studies. *The Lancet Neurology* **18**, 1091-1102 (2019).

5 Foo, J. N. *et al.* Identification of risk loci for Parkinson disease in Asians and comparison of risk between Asians and Europeans: a genome-wide association study. *JAMA neurology* **77**, 746-754 (2020).

6 Loesch, D. P. *et al.* Characterizing the genetic architecture of Parkinson's disease in Latinos. *Annals of Neurology* **90**, 353-365 (2021).

7 Kia, D. A. *et al.* Identification of candidate Parkinson disease genes by integrating genome-wide association study, expression, and epigenetic data sets. *JAMA neurology* **78**, 464-472 (2021).

8 Pan, H. *et al.* Genome-wide association study using whole-genome sequencing identifies risk loci for Parkinson’s disease in Chinese population. *npj Parkinson's Disease* **9**, 22 (2023).

9 Stark, C. *et al.* BioGRID: a general repository for interaction datasets. *Nucleic acids research* **34**, D535-D539 (2006).

10 Keshava Prasad, T. *et al.* Human protein reference database—2009 update. *Nucleic acids research* **37**, D767-D772 (2009).

11 Martin, A. *et al.* BisoGenet: a new tool for gene network building, visualization and analysis. *BMC bioinformatics* **11**, 1-9 (2010).

12 Lin, M. *et al.* Integrative transcriptome network analysis of iPSC-derived neurons from schizophrenia and schizoaffective disorder patients with 22q11. 2 deletion. *BMC systems biology* **10**, 1-20 (2016).

13 Love, M. I., Anders, S., Kim, V. & Huber, W. RNA-Seq workflow: gene-level exploratory analysis and differential expression. *F1000Research* **4** (2015).

14 Taskesen, E. pca: A Python Package for Principal Component Analysis. (2020).

15 Wall, M. E., Rechtsteiner, A. & Rocha, L. M. Singular value decomposition and principal component analysis. *A practical approach to microarray data analysis*, 91-109 (2003).

16 Metsalu, T. & Vilo, J. ClustVis: a web tool for visualizing clustering of multivariate data using Principal Component Analysis and heatmap. *Nucleic acids research* **43**, W566-W570 (2015).

17 Uhlén, M. *et al.* Tissue-based map of the human proteome. *Science* **347** (2015).

18 Hawrylycz, M. J. *et al.* An anatomically comprehensive atlas of the adult human brain transcriptome. *Nature* **489**, 391 (2012).

19 Consortium, G. The Genotype-Tissue Expression (GTEx) pilot analysis: Multitissue gene regulation in humans. *Science* **348**, 648-660 (2015).

20 Rouillard, A. D. *et al.* The harmonizome: a collection of processed datasets gathered to serve and mine knowledge about genes and proteins. *Database* **2016** (2016).

21 Li, J. *et al.* Integrated systems analysis reveals a molecular network underlying autism spectrum disorders. *Molecular systems biology* **10**, 774 (2014).

22 Bader, G. D. & Hogue, C. W. An automated method for finding molecular complexes in large protein interaction networks. *BMC bioinformatics* **4**, 1-27 (2003).

23 Consortium, G. O. The Gene Ontology (GO) database and informatics resource. *Nucleic acids research* **32**, D258-D261 (2004).

24 Liao, Y., Wang, J., Jaehnig, E. J., Shi, Z. & Zhang, B. WebGestalt 2019: gene set analysis toolkit with revamped UIs and APIs. *Nucleic acids research* (2019).

25 Shannon, P. *et al.* Cytoscape: a software environment for integrated models of biomolecular interaction networks. *Genome research* **13**, 2498-2504 (2003).

26 Kanehisa, M. *et al.* KEGG for linking genomes to life and the environment. *Nucleic acids research* **36**, D480-D484 (2007).

27 Lake, B. B. *et al.* Integrative single-cell analysis of transcriptional and epigenetic states in the human adult brain. *Nature biotechnology* **36**, 70-80 (2018).

28 Greenwood, T. A. *et al.* Genome-wide association of endophenotypes for schizophrenia from the Consortium on the Genetics of Schizophrenia (COGS) study. *JAMA psychiatry* **76**, 1274-1284 (2019).

29 Farrell, M. *et al.* Evaluating historical candidate genes for schizophrenia. *Molecular psychiatry* **20**, 555-562 (2015).

30 Blauwendraat, C., Nalls, M. A. & Singleton, A. B. The genetic architecture of Parkinson's disease. *The Lancet Neurology* **19**, 170-178 (2020).

31 Holland, S. M. Principal components analysis (PCA). *Department of Geology, University of Georgia, Athens, GA*, 30602-32501 (2008).

32 Bloem, B. R., Okun, M. S. & Klein, C. Parkinson's disease. *Lancet* **397**, 2284-2303 (2021). <https://doi.org:10.1016/s0140-6736(21)00218-x>

33 Athauda, D. & Foltynie, T. Insulin resistance and Parkinson’s disease: a new target for disease modification? *Progress in neurobiology* **145**, 98-120 (2016).

34 Chinta, S. J. *et al.* Cellular senescence is induced by the environmental neurotoxin paraquat and contributes to neuropathology linked to Parkinson’s disease. *Cell reports* **22**, 930-940 (2018).

35 Shackman, A. J. & Fox, A. S. (Am Psychiatric Assoc, 2021).

36 Kobrynski, L. J. & Sullivan, K. E. Velocardiofacial syndrome, DiGeorge syndrome: the chromosome 22q11. 2 deletion syndromes. *The Lancet* **370**, 1443-1452 (2007).

37 Nalls, M. A. *et al.* Large-scale meta-analysis of genome-wide association data identifies six new risk loci for Parkinson's disease. *Nature genetics* **46**, 989-993 (2014).

38 Alanis-Lobato, G., Andrade-Navarro, M. A. & Schaefer, M. H. HIPPIE v2. 0: enhancing meaningfulness and reliability of protein–protein interaction networks. *Nucleic acids research*, gkw985 (2016).
